# Innovative α-MnO_2_/Nanocarbon Ball Additive for Enhancing the Molecular Structure, Emission Control, and Engine Performance of Diverse Biodiesel Generations

**DOI:** 10.1021/acsomega.3c05848

**Published:** 2024-01-25

**Authors:** Ahmad Yousefvand, Mehdi Ardjmand, Hamidreza Mogadamzadeh

**Affiliations:** † Department of Chemical Engineering, South Tehran Branch, Islamic Azad University, Tehran 1584743311, Iran; ‡ Nanotechnology Research Center, South Tehran Branch, Islamic Azad University, Tehran 1584743311, Iran

## Abstract

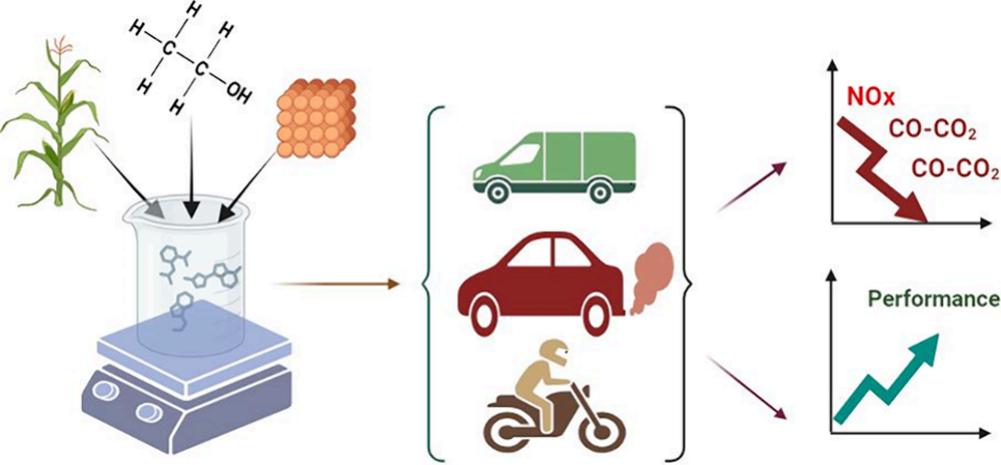

This study investigates the utilization of an α-MnO_2_/nanocarbon ball (NCB) additive to enhance the performance of second-, third-, and fourth-generation biodiesels (SSGB, PVB, and GMCB). Various tests including XRD, XPS, TEM, HRTEM, BET, torque and power measurements, EGT, BTE, emissions analysis (CO_2_, CO, HC, soot, and NOx), and BSFC were conducted. The combination of GMCB5N50 with α-MnO_2_/NCB yielded the highest torque (35.77 N m) and power (6.47 kW), indicating an improved engine performance. GMCB5N50 exhibited efficient combustion with a peak pressure of 76.04 bar. The nanoadditive also demonstrated significant reduction in BSFC, achieving up to 34% improvement in fuel efficiency. When GMCB20N50 was used, the highest BTE values were observed, reaching approximately 39.5%. EGT values for GMCB5N50 were only slightly elevated compared to pure diesel. Notably, GMCB20N50 showcased substantial decreases in emissions, including carbon dioxide (CO_2_: 55% reduction), carbon monoxide (CO: 35% reduction), hydrocarbons (HC: 58% reduction), and soot (98% reduction), indicating a promising direction for the development of low-emission alternative fuels. The investigation of the effects of the oxygen lattice, surface area, and oxygen adsorption on engine performance and emission reduction revealed their positive contributions. These findings highlight the potential of the studied α-MnO_2_/NCB additive for improving biodiesel performance and advancing the development of sustainable and environmentally friendly fuels.

## 1 Introduction

Countries worldwide are currently grappling with the challenges posed by a considerable shift in energy demand and rapid expansion of the population. These transformations have led to widespread resource scarcity, highlighting the growing significance of alternative energy sources.^1^ Furthermore, it is important to acknowledge that excessive utilization of conventional energy resources is associated with environmental degradation and pollution. Such pollution not only poses risks to human health within communities but also has detrimental effects on ecosystems. The adoption of biofuels as a renewable energy source presents a potential solution to mitigate and address these challenges, offering long-term benefits in terms of sustainability and environmental preservation.^2^

Multiple generations of biofuels exist, encompassing the first, second, third, and fourth iterations. The utilization of first-generation biofuels may potentially trigger a food crisis, despite their wide availability across various nations.^3^ Several producer nations have embraced the utilization of second-generation biofuels, although this generation is constrained by certain limitations, such as its restricted adaptability to diverse climatic conditions.^4^ In the context of biofuel production, the third generation presents greater complexity in terms of production processes and oil extraction yet remains a viable option. Among the various generations, the fourth generation holds particular significance. It is noteworthy that the first generation possesses traits that align with those of both the second and third generations. Introducing genetic modifications in the first and second generations emerges as an intriguing approach, as it has the potential to not only enhance yields but also confer resilience against adverse climatic conditions onto the crops.^5^ According to the study conducted by Jafarihaghighi et al., the fourth generation of biodiesel was found to exhibit lower effectiveness compared with other generations. However, the incorporation of nanoadditives has been identified as a potential solution to enhance the efficiency of the fourth-generation biodiesel to meet desired levels.^6^

Nanoadditives (NAs), including alumina and multiwalled carbon nanotubes, have been utilized in various applications to enhance performance and mitigate the emission of hazardous gases. Tomar and Kumar investigated the impact of these NAs on engine performance and exhaust emissions. The findings indicated that the presence of NAs resulted in an improvement in brake thermal efficiency (BTE) by approximately 2–13% and a reduction in exhaust emissions by approximately 5–60%. In comparing alumina and carbon nanotubes, it was observed that alumina demonstrated greater effectiveness in this context.^7^ In a study of Badawy et al., carbon nanoadditives (NAs) were employed, and their efficacy was assessed for the second-generation biodiesel. The study findings indicated that the incorporation of carbon NAs into Jatropha biodiesel resulted in a reduction in engine emissions, including carbon monoxide (CO), nitrogen oxides (NOx), and hydrocarbons (HC). Furthermore, the utilization of NAs led to decreased values of exhaust gas temperature (EGT) and brake-specific fuel consumption (BSFC) compared with standard diesel fuel. These results highlight the substantial impact of nanoadditives on both engine performance and emissions.^8^ According to Gad et al., carbon nanotubes (CNTs) and graphene nanosheets were employed to assess the engine efficiency and emissions. The introduction of a concentration of 100 ppm of these nanomaterials led to a substantial reduction in smoke emissions, accompanied by a decrease of approximately 27–28% in CO and NOx levels. Furthermore, the utilization of graphene nanosheets at the same concentration exhibited superior combustion and performance characteristics compared to CNTs.^9^ In a study conducted by Ramakrishnan et al., CNTs were utilized to assess their efficacy in reducing emissions. The nanoparticles (NPs) were applied at concentrations of 100 and 50 ppm (ppm) to mitigate the emissions. However, the findings revealed that the reduction in NOx, CO, HC, and smoke emissions achieved by CNTs was only in the range of 5.2–9.2%. These results indicated a lower emission reduction yield compared to previous studies.^10,11^ Hosseinzadeh-Bandbafha et al. investigated the impact of carbon nanoparticles on the engine performance in their study. The incorporation of carbon nanoparticles into water-emulsified biodiesel (WEB) resulted in improved thermal efficiency and brake power (BP) while reducing brake-specific fuel consumption (BSFC). Specifically, the emulsified fuel blend containing 38 M carbon nanoparticles exhibited enhancements in BP and brake thermal efficiency by approximately 1.07 kW and 11.58%, respectively, accompanied by a reduction in BSFC by around 107.3 g/kWh. However, the inclusion of carbon nanoparticles in WEB led to increased HC and CO emissions due to the higher carbon content in the fuel blend, although NOx emissions decreased. Despite these effects, the utilization of carbon nanoparticles in WEB mitigated the negative impact of water on fuel economy by positively influencing thermal efficiency.^12^ The study shows that adding multiwalled carbon nanotubes (MWCNTs) to a mix of 20% palm-oil biodiesel and fossil diesel (B20) significantly boosts diesel engine performance by reducing ignition delay, advancing combustion timing, and shortening combustion duration. This enhances fuel efficiency and thermal efficiency but raises NOx emissions while lowering CO and unburned hydrocarbon (UHC) emissions. Notably, B20M100, with the highest MWCNT concentration, is found to be less effective due to MWCNT clumping. The study highlights the need for further research on MWCNTs’ impact on fuel injection system wear, potential nanoparticle emissions from engine exhaust, and a deeper understanding of MWCNTs in diesel engine combustion chambers.^13^ The study investigated how titanium dioxide (TiO_2_) nanoparticles and ethanol impact a diesel engine’s performance and emissions using various fuel blends. It was found that at low loads, fuel consumption decreased by 5% but it increased by up to 17% at higher loads, with a 6% improvement in thermal efficiency. TiO_2_ additives improved engine performance, reducing CO emissions by 30% and HC by 6–21%, while lowering CO_2_ levels and smoke opacity compared to pure diesel. However, a higher ethanol content in the blends led to an 18–70% increase in NOx emissions, especially at higher loads. The study suggests that maintaining TiO_2_ nanoparticles at a concentration of 100 ppm enhances the overall engine performance and emission characteristics, making blended fuels of diesel, biodiesel, and ethanol a viable alternative to pure diesel. Future research may explore other nanoparticles for further optimization.^14^ In this study, an advanced and novel approach is employed to enhance engine efficiency and reduce pollution by utilizing carbon-based nanoadditives. The focus is on the utilization of nanocarbon balls (NCBs) and α-MnO_2_ as a unique combination to achieve significant improvements in both engine performance and emission control. The novelty lies in the exploration of third and fourth generation biodiesels, which offer promising characteristics for sustainable fuel sources. The fourth-generation biodiesel, in particular, demonstrates enhanced adaptability to harsh weather conditions and exhibits a higher yield potential while requiring reduced water usage. The inclusion of NCBs in the study considers various factors such as double bonds, carbon chain lengths (CCLs), saturated and unsaturated acids, and parameters like the O/C and H/C ratios to comprehensively evaluate their effects. Furthermore, the effects of the oxygen lattice, oxygen adsorption, *d* space (lattice spacing), and surface area of the nanoadditives are investigated in the context of engine performance and emission reduction. By exploring the novel combination of NCBs and α-MnO_2_, along with investigating the effects of oxygen lattice, oxygen adsorption, *d* space, and surface area, this study aims to provide valuable insights into the advancement of sustainable and efficient engine technologies while mitigating environmental pollution.

## 2 Materials and Methods

### 2.1 Preparation of Biodiesels

The biodiesels selected for this study encompass second-, third-, and fourth-generation varieties, namely, sweet-scented geranium biodiesel (SSGB), *Pyropia vietnamensis* biodiesel (PVB), and genetically modified canola biodiesel (GMCB). The production of SSGB involved mechanical methods, which entailed the separation of grains from impurities and shells during the preparation stage. Thermal processes were employed to facilitate the oil lubrication within the grains. Mechanical lubrication was carried out using box presses and extraction wheels, performed by the Armaghan Meymand Company. The PVB species belongs to the Bangiaceae family of red algae and is typically found in shallow water and intertidal regions. During the purification process, additional materials were eliminated. To facilitate processing, PVB was dried for a period of 4–5 days followed by pulverization. Lipid extraction from PVB involved multiple cycles of crushing in the presence of a solvent blend consisting of isopropyl alcohol and hexane. The resulting lipid was subsequently separated from the biomass, and the solvent was evaporated through heating. This product was prepared by Shirin Rayan. As depicted in Figure S1, the initial step in biofuel generation mirrored.

In this research, the production of biodiesel was carried out through transesterification using a KOH-catalyzed reaction. The transesterification procedure involved a methanol-to-oil ratio ranging from 1 to 3 (v/v). For the dissolution of 2.2 g of KOH, 130 mL of methanol was utilized. The exothermic nature of the KOH dissolution reaction caused the alcohol to evaporate, which was then condensed and returned to the solution. Once the catalyst was completely dissolved, the methoxide was added to the oil and the mixture was heated at 60 °C for 1 h. To remove impurities, the resulting mixture was washed with warm distilled water. The volume of water used for each washing step was twice that of biodiesel. After multiple washes, the biodiesel and water phases were separated by using a separation funnel or centrifuge. The separated biodiesel was subjected to a settling process at a temperature of 50 °C, allowing the water to separate and be discharged through a lower valve. The acid value of biodiesel was measured in the final phase. The viscosity and density characteristics were determined by using a Stabinger Viscometer (Anton Paar Co., Austria), specifically the SVM3000 model. Gas chromatography (GC) analysis (Claus 580 GC model, PerkinElmer Co., USA) was employed to identify the compounds present in biodiesel generated through the transesterification process. For GC analysis, 0.1 g of biodiesel and a diluted solution of an internal standard were prepared in a solvent. The resulting mixture was injected into the gas chromatography system, and the detector response provided concentration data for the compounds present in the sample. The chromatograms obtained from the detector responses showed peaks corresponding to the concentrations of different compounds including methylated fatty acids and internal standards. The area under each peak represented the concentration of the corresponding compound in the sample. Additionally, the cetane number (CN) was measured by using an Octan-IM device. The synthesis of α-MnO_2_/NCBs, a carbon-based nanoadditive, was carried out through an incomplete combustion method, resulting in the formation of carbon nanoballs. These processes and characterization of the samples are described in detail in the Supporting Information. Fuel samples were then prepared by combining the biodiesels with different concentrations of the nanoadditive, and the testing procedures were conducted using an engine and dynamometer setup, as outlined in the Supporting Information. The sections in the Supporting Information provide comprehensive details of the experimental procedures, instrumentation, and analysis conducted in this study.

The net biodiesels (second, third, and fourth generation) were blended with diesel fuel in varied combinations (B5, B10, and B20). New fuels are named SSGB5N50 (50 ppm concentration), SSGB10N50, SSGB20N50, and SSGB5N100 (100 ppm concentration), SSGB10N100, SSGB20N100, PVB5N50, PVB10N50, PVB20N50, PVB5N100, PVB10N100, PVB20N100, GMCB5N50, GMCB10N50, GMCB20N50, GMCB5N100, GMCB10N100, and GMCB20N100. An original engine was employed because the price is too high and is not employed in the industry. The viscosity, density, and heating value features are indicated in Tables S1, S2, and S3.

### 2.2 Engine Characteristics

In this report, Table 1 provides information about the engine and dynamometer utilized in the study. The dynamometer is connected to the motor through a guard shaft, which is enclosed in a steel enclosure for safety purposes. The dynamometer employs a magnetic field to automatically measure torque and a magnetic sensor to calculate rotational speed. It then computes motor power and displays the results in the form of data or graphs. A force gauge connected to the dynamometer assesses the load on the motor axis as the load increases. Motor speed is measured using a magnetic sensor, while several temperature sensors are installed on the engine to measure parameters such as engine oil temperature, engine outlet water temperature, exhaust gas temperature, engine fuel flow, and air manifold pressure. Additionally, the test chamber is equipped with temperature and ambient pressure sensors, and a temperature sensor is mounted on the dynamometer to monitor its instantaneous temperature.

**Table 1:** Specifications of the Diesel Engine Used in the Test

specification	explanations
model	3LD510
number of cylinders	1
bore and stroke (mm)	85 × 90
cycle	4 strokes
aspiration	naturally aspirated
displacement (cm^3^)	510
rotation	counter-clockwise (view from main PTO side)
cooling system	air
combustion system	direct injection
oil sump capacity (L)	1.75
fuel tank capacity (L)	5.3
width (mm)	422
length (mm)	466
height (mm)	568
compression ratio	17.5:1
dry weight (kg)	60
cylinder diameter (mm)	85
cylinder course (mm)	90
cylinder volume (cm^3^)	510
maximum power hp (3000 rpm)	12.2
maximum torque 1800 rpm (Nm)	33

For contamination evaluation, the MAHA-MGT5 instrument is employed. The engine speed is adjusted accordingly, and a probe is positioned within the embedded chamber at the exhaust outlet. After a 20 s interval, the contaminant value stabilizes in the computer system, and the measurement is recorded. Table 2 presents the specifications of the examinations, which were conducted three times with a significance level (*P* value) of approximately 0.05. The maximum torque is considered to be a key performance parameter for the engine, as indicated in Table 3, where the boundary conditions and input are described.

**Table 2:** MAHA-MGT5 Analyzer Specifications Employed in the Test

specification	explanations
measuring principle electrochemical detection	O_2_, NO
measuring principle infrared spectrometry	HC, CO, CO_2_
measurable gases	HC, CO, CO_2_, O_2_, NO (option)
flow	3.5 L/min
warm-up time	480 s
accuracy class	O (OIML)
working pressure	0.75–1.1 bar
power supply	1/N/PE 85 V/285 V 50 Hz
on-board voltage	12 V/42 V
dimensions total (*L* × *W* × *H*)	240 mm × 560 mm × 300 mm
weight	10 kg
CO_2_ measurement range/measured value resolution (max.)	0–20% vol/0.01
CO measurement range/measured value resolution (max.)	0–15% vol/0.01
O_2_ measurement range/measured value resolution (max.)	0–25% vol/0.01
HC measurement range/measured value resolution (max.)	0–9999 ppm/0.1 (Hexan), 0–20000 ppm/1 (Propan)
NO (option) measurement range/measured value resolution (max.)	0–5000 ppm/1
lambda (calculated)	0.5–9.99/0.01

**Table 3:** Determined Preliminary and Boundary Circumstances

specifications	descriptions
engine speed (EA)	1800 rpm
air inlet temperature	293.15 K
fuel injection temperature	330.15 K
air inlet pressure	1 bar
cylinder wall temperature	475.15 K
cylinder head temperature	575.15 K
fuel consumption Urtica	2.1 L/h
fuel injection pressure	200 bar

## 3 Results and Discussion

### 3.1 Nanoadditive Characterization

#### 3.1.1 XRD Pattern

**Figure 1:**
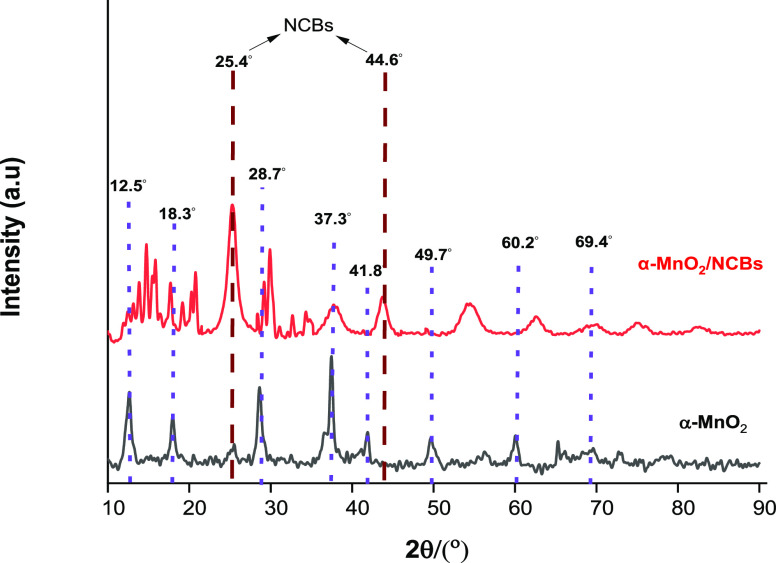
XRD pattern of α-MnO_2_ and α-MnO_2_/NCBs.

The X-ray diffraction (XRD) analysis of α-MnO_2_ revealed distinct diffraction peaks at specific angles, indicating the crystallographic planes present in the sample. The diffraction peaks were observed at 12.6, 18.1, 28.6, 37.4, 41.9, 49.6, 60.1, and 69.6°, corresponding to the crystal planes (110), (200), (310), (211), (301), (411), (521), and (541), respectively. The XRD pattern obtained for α-MnO_2_ is depicted in Figure 1 (reference: 96-151-4117).^15−17^ The XRD analysis of α-MnO_2_/NCBs showed reduced intensity or overlapping peaks compared to the diffraction pattern of pure α-MnO_2_. This suggests a lower concentration or content of α-MnO_2_ in the α-MnO_2_/NCBs samples.^18^ The nanocrystal size was determined via the Scherrer equation (eq 1)
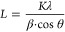
1

The crystal size of α-MnO_2_ was estimated to be 25 nm using the Scherrer equation, which takes into account factors such as X-ray wavelength, full width at half-maximum (fwhm), and the Bragg angle.^19^

#### 3.1.2 Morphology

**Figure 2:**
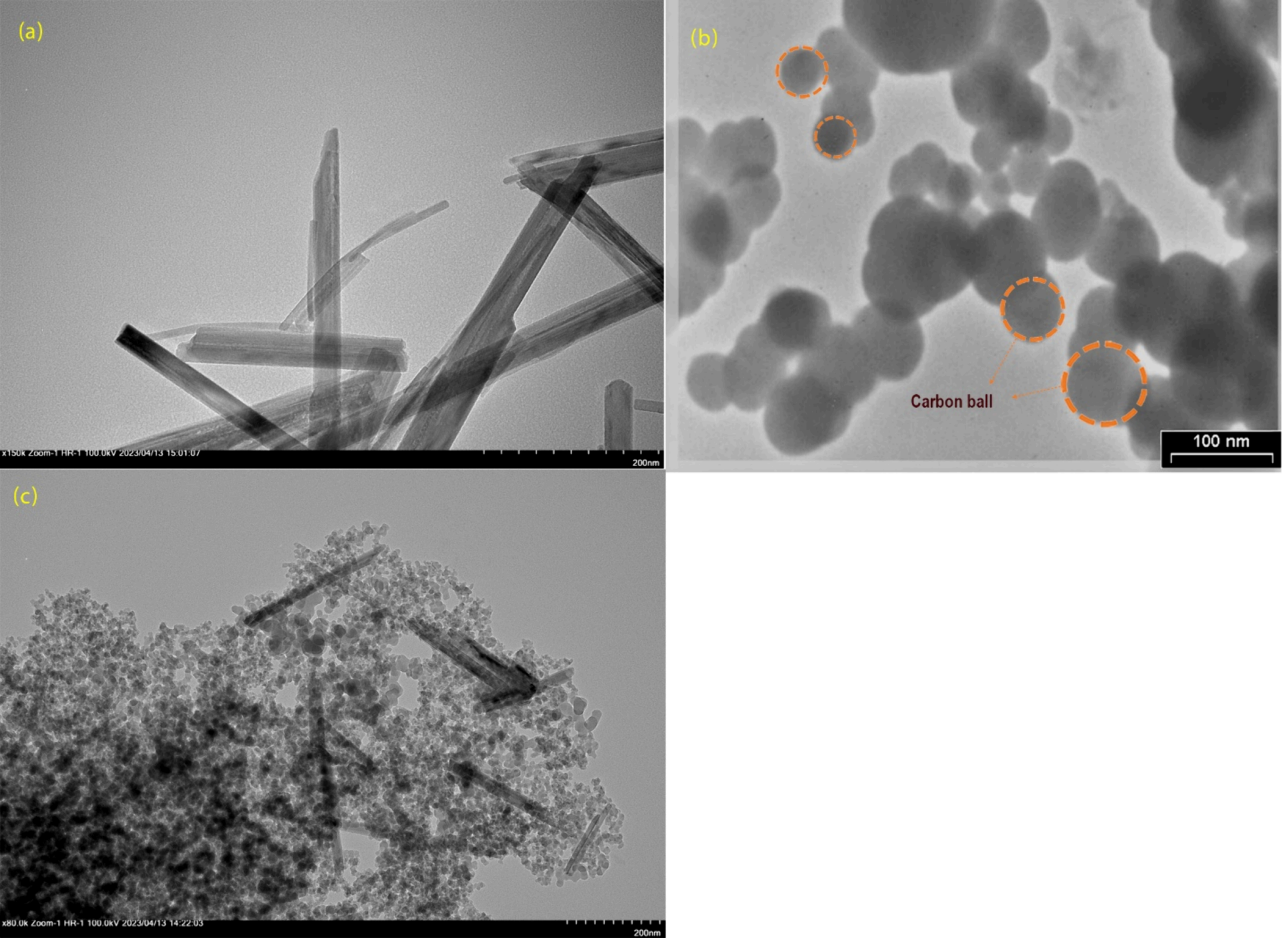
TEM of (a) α-MnO_2_ (200 nm), (b) NCBs (100 nm), and (c) α-MnO_2_/NCBs (200 nm).

TEM images were captured to examine the morphology and structure of the additive, namely, α-MnO_2_, NCBs,^20^ and α-MnO_2_/NCBs (as illustrated in Figure 2).^21^ The TEM images of the α-MnO_2_ additive, both unsupported and supported on NCBs, were analyzed to confirm their morphology and formation, as depicted in Figure 2.^17^ TEM analysis indicates that the majority of the carbon accumulation consisted of spherical substances, with their diameters varying between 20 and 50 nm.^17^ The produced α-MnO_2_ displays an average diameter between 10 and 50 nm and lengths that stretch from 0.1 to 2 μm.^22^ The pure α-MnO_2_ exhibited a nanorod structure with a smooth surface and an even distribution. The presence of α-MnO_2_ on the surfaces of the NCB support was observed in the TEM images of α-MnO_2_ supported on NCBs,^17^ indicating the successful formation and dispersion of the additive. The dispersion of the additive on the support is of great significance in process as it can impact surface area, accessibility, kinetics, selectivity, and stability.^23^

**Figure 3:**
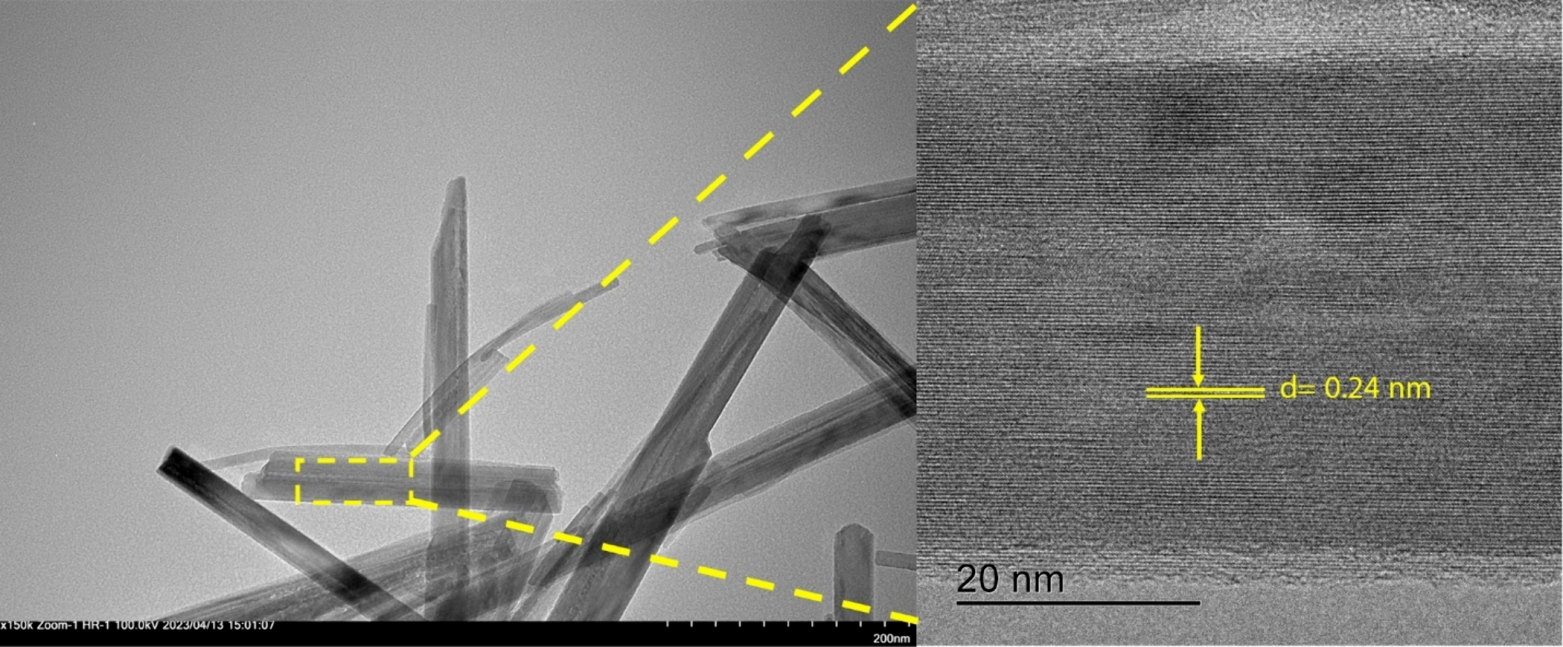
HRTEM of α-MnO_2_ at 200 and 20 nm.

High-resolution transmission electron microscopy (HRTEM) was utilized to determine the crystal facets exposed on the side walls of the α-MnO_2_ nanorod (refer to Figure 3). The HRTEM analysis revealed a lattice distance of 0.24 nm along the growth axis, indicating the presence of the (310) facet of α-MnO_2_. Consequently, it can be inferred that the perpendicular side walls also consist of the (310) facet.^24^ In the case of α-MnO_2_ with the major exposed facet, the diffraction peak corresponding to (310) exhibits the highest intensity compared to other diffraction peaks.^25^ The successful manipulation of synthesizing α-MnO_2_ with predominant high-index exposed facets resulted in a substantial enhancement in additive activity.^25^

#### 3.1.3 BET Analysis

Table 4 displays the surface area and pore volume characterizations of α-MnO_2_, α-MnO_2_/NCBs, and NCBs. The findings revealed that pure α-MnO_2_ exhibited a relatively low surface area and pore volume. Nevertheless, the introduction of support materials led to a remarkable augmentation in both the surface area and pore volume. The inclusion of support materials facilitated an expanded surface area, enabling enhanced interactions between the active material and the reactants.^26^

**Table 4:** Surface Area and Pore Volume of the NCBs, α-MnO_2_/NCBs,and α-MnO_2_ Materials

samples	*S*_BET_ (m^2^/g)	pore volume (cm^3^/g)
α-MnO_2_	47	0.139
NCBs	635	0.479
α-MnO_2_/NCBs	235	0.385

#### 3.1.4 XPS Analysis

**Figure 4:**
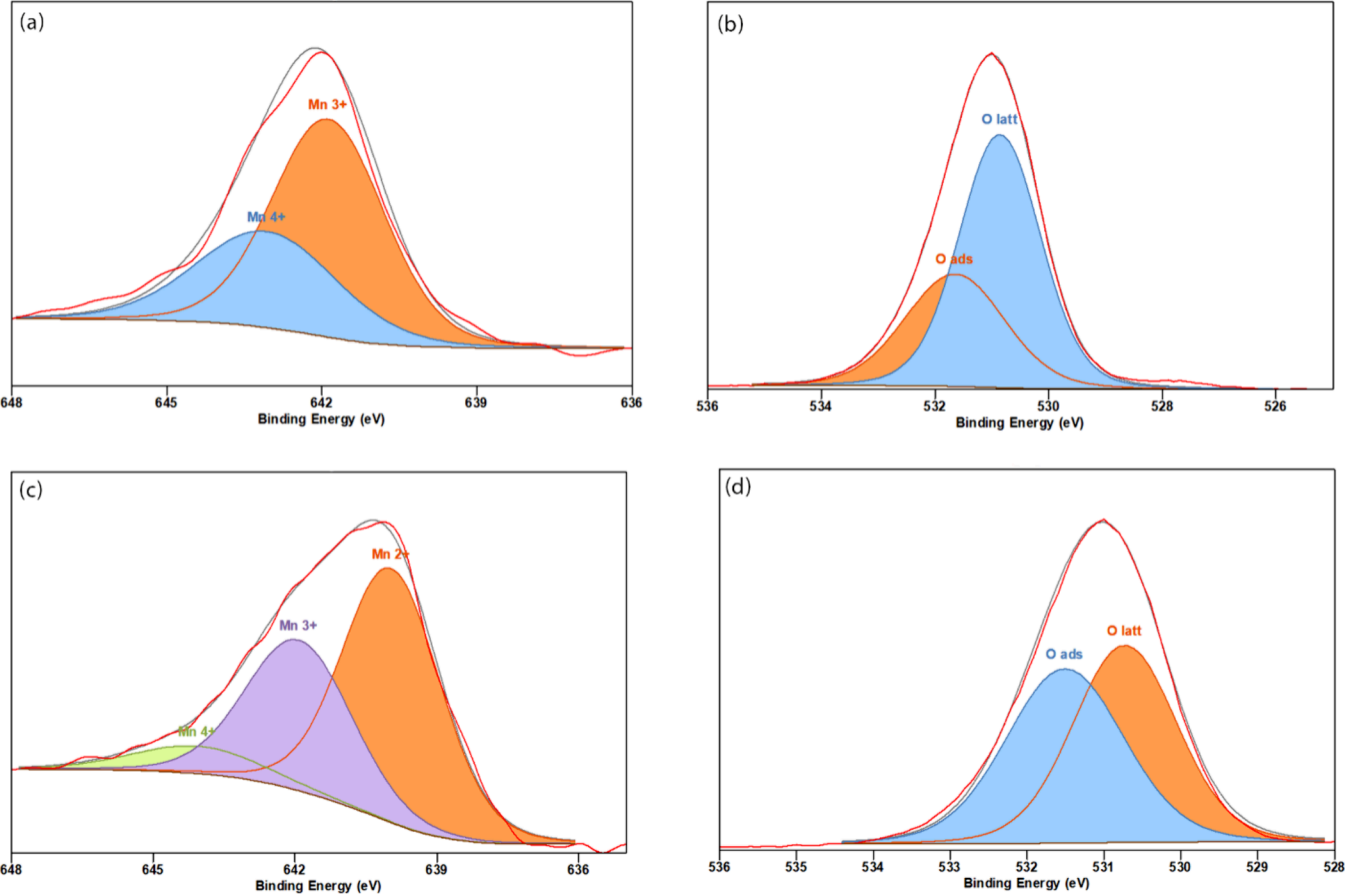
XPS spectra of α-MnO_2_ ((a) Mn 2p3/2 and (b) O 1s) and α-MnO_2_/NCBs ((c) Mn 2p3/2 and (d) O 1s).

XPS analysis was employed to investigate the elemental composition of the additive and its relationship to additive performance. The XPS results revealed the presence of Mn 2p3/2 and O 1s peaks for α-MnO_2_ and α-MnO_2_/NCBs, as shown in Figure 4. The Mn 2p3/2 peak exhibited three distinct peaks corresponding to different oxidation states of Mn in α-MnO_2_. The Mn^2+^, Mn^3+^, and Mn^4+^ peaks were observed at energy ranges of approximately 639–640, 641–642, and 642–644 eV, respectively.^27,28^Table S4 shows the area of Mn^2+^, Mn^3+^, Mn^4+^, Mn^3+^/Mn^4+^, Mn^2+^/Mn^3+^, O _latt%_, O _ads%_, and O _latt%_/ O _ads_%. The incorporation of a support material in additive ozonation has the potential to induce the formation of a reduced oxidation state, specifically Mn^2+^. As shown in Table S4, the order of the Mn^2+^/Mn^3+^ ratio was not observed for α-MnO_2_. The presence of the support material serves as an electron acceptor, facilitating the transfer of electrons from the additive species and promoting the reduction of these species to lower oxidation states.^29^ The reduced oxidation states observed in the additive species can exhibit enhanced reactivity in generating the O_ads_ and the O_latt_ species. The presence of a support material plays a crucial role in stabilizing the reduced state of the additive species, preventing its reversion to a higher oxidation state. This stabilization mechanism involves the facilitation of electron transfer, facilitated by the high electron affinity of the support material and the high electron conductivity of the additive species.^30^

The XPS analysis of α-MnO_2_/NCBs revealed two distinct peaks in the O 1s spectrum. The first peak corresponds to surface-adsorbed oxygen species (O_ads_), and the second peak represents lattice oxygen (O_latt_). The energy ranges for these peaks were approximately 530–532 and 528–530 eV, respectively.^31^ The oxidation state of Mn in a nanoadditive influences the concentration of oxygen vacancies and the reactivity of lattice oxygen. The reduction of Mn to lower oxidation states promotes the formation of oxygen vacancies, which are highly reactive sites capable of enhancing the additive activity of the material.^32^ The oxidation state of Mn can also affect the reactivity of the lattice oxygen in the nanoadditive. Lowering the oxidation state of Mn results in an increase in the electron density of the additive, which can facilitate the removal of oxygen atoms from the lattice and create oxygen vacancies.^33,34^

#### 3.1.5 Fatty Acids (FAs)

**Figure 5:**
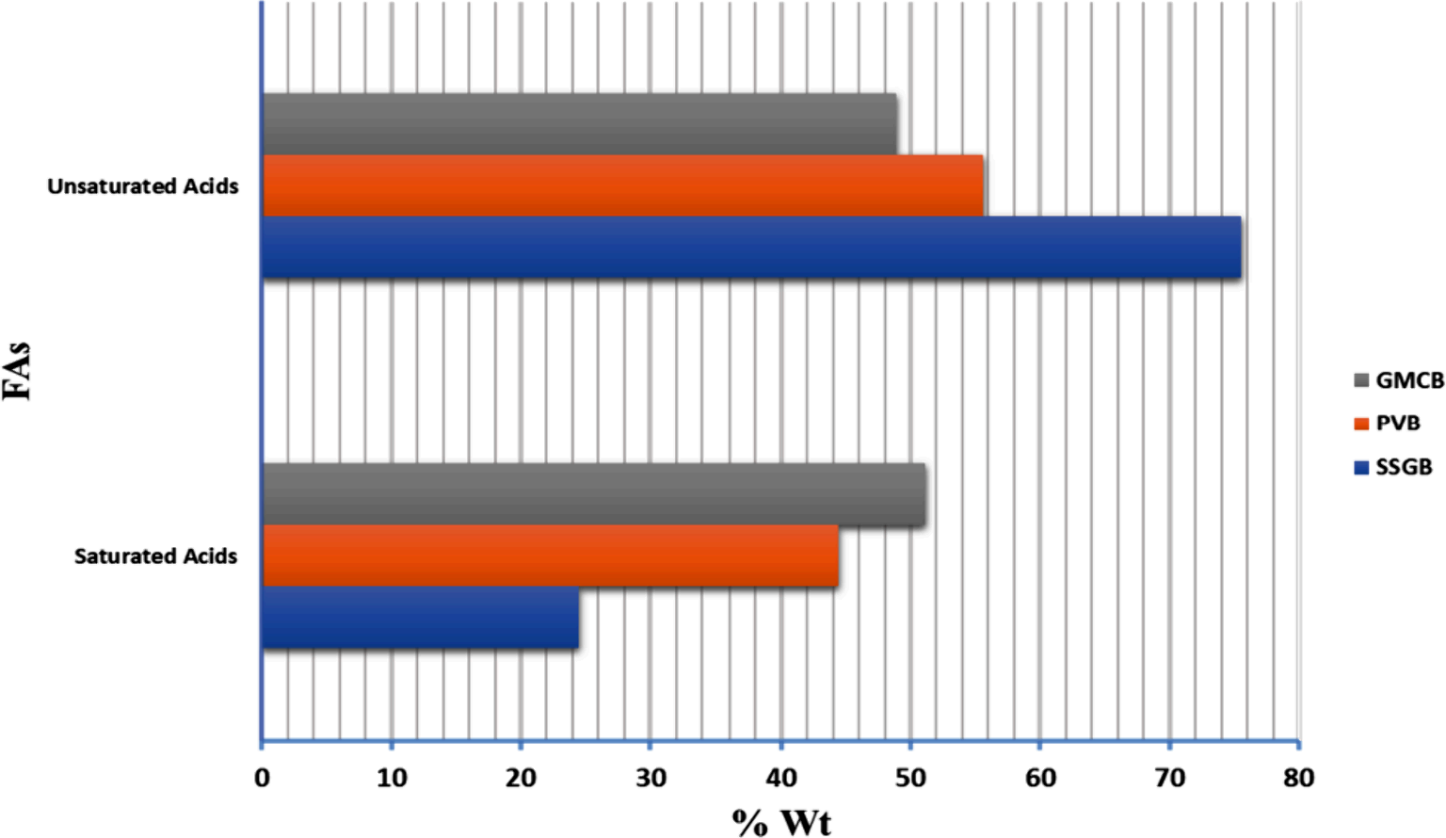
Comparing the saturated and unsaturated acids for various samples.

A comprehensive overview of the fatty acid (FA) composition can be found in Table S5. The structure of FAs significantly influences the various characteristics of biodiesel. According to Table S5, GMCB, PVB, and SSGB exhibited the highest levels of saturated acids, while SSGB, PVB, and GMCB showed the highest levels of unsaturated acids (Figure 5). For GMCB, the predominant saturated acid was palmitic acid (39.75% wt), with the lowest being arachidic acid (0.02% wt). The highest unsaturated acids in GMCB were oleic acid (23.65% wt), while the lowest level was observed for linolenic acid (1.27% wt). Conversely, SSGB had a lower percentage of saturated acids (24.49% wt), with palmitic acid being the most abundant. However, SSGB exhibited higher levels of unsaturated acids with oleic acid being the predominant component. The presence of saturated and unsaturated acids in biodiesel can significantly impact the engine performance and exhaust gas properties. The increase in saturated acids and longer CCLs can influence properties such as viscosity, heating value (HV), and CN. Among the samples, GMCB exhibited the longest CCLs and highest levels of saturated acids, which could affect the viscosity, HV, and CN of the biodiesel. Therefore, the utilization of fourth-generation biodiesel in combination with NCBs at a 5% diesel blend ratio led to improved CN, HV, and viscosity properties. Furthermore, the spherical structure of NCBs may have contributed to a reduction in viscosity due to their enhanced mobility, while the longer carbon chain length could be attributed to the higher carbon content. The increased presence of saturated acids may lead to elevated oxygen-to-carbon ratio (O/C) and hydrogen-to-carbon ratio (H/C). On the other hand, the presence of double bonds can result in a decrease in O/C and H/C ratios. These factors play a role in influencing the properties of exhaust gases and engine performance. In this study, GMCB exhibited the highest H/C and O/C ratios among the tested biodiesels.^35^

### 3.2 Performance and Emission Analysis

#### 3.2.1 Combustion

**Figure 6:**
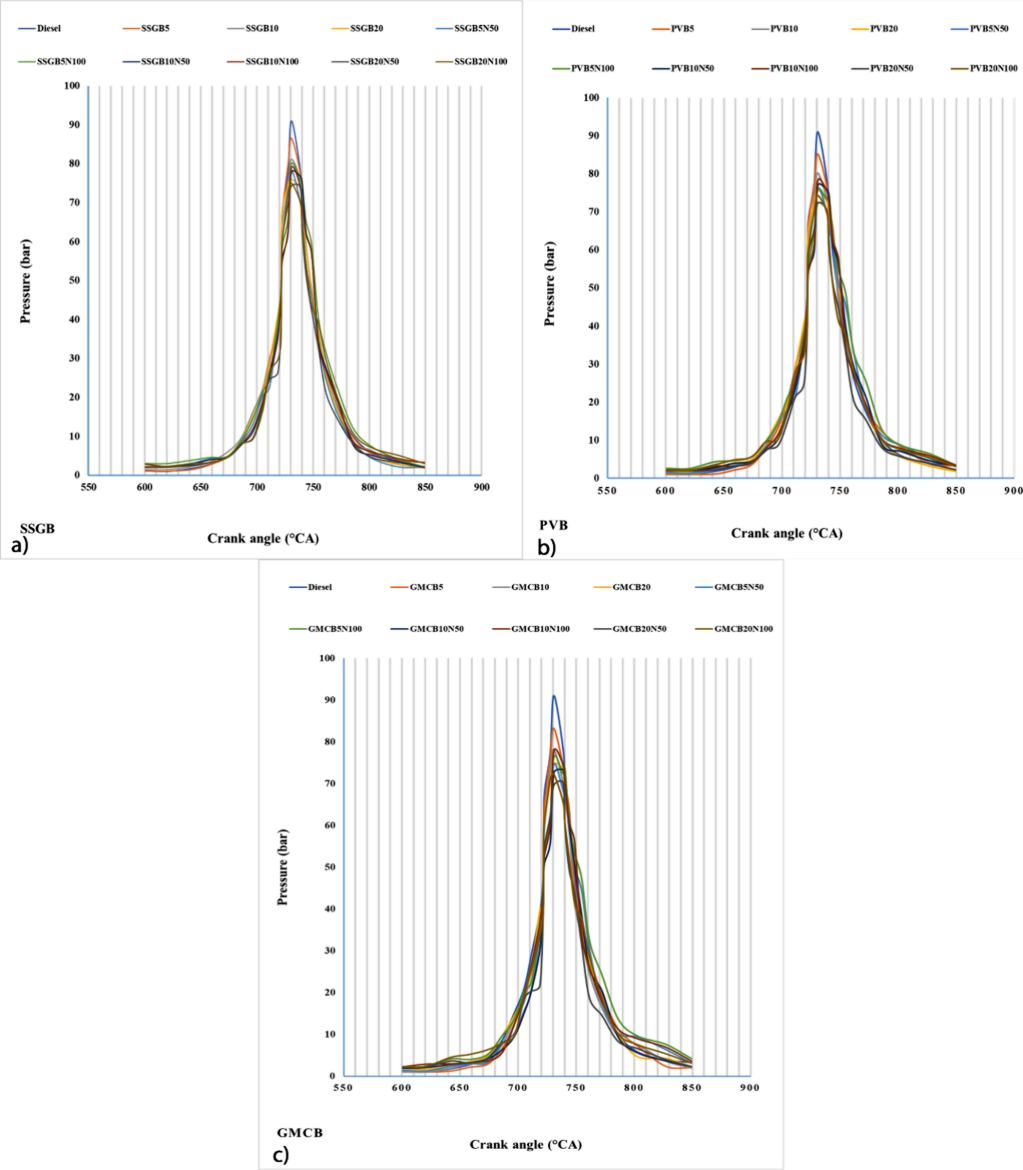
Value of pressure for different levels of biodiesel and nanoadditive for (a) sweet-scented geranium biodiesel (SSGB), (b) *Pyropia vietnamensis* biodiesel (PVB), and (c) genetically modified canola biodiesel (GMCB).

Figure 6 illustrates the values of the cylinder pressure and crank angle for samples. In relation to peak pressures, the fourth generation of biodiesel exhibited the lowest values, while the second generation showed the highest peak pressures. The incorporation of biofuel into diesel resulted in a reduction in peak pressures across all biodiesel types. Biodiesel contains oxygen in its molecular structure due to the presence of ester groups. The oxygen content in biodiesel promotes a more complete combustion, but it can also result in a slower and more controlled combustion process. This slower combustion rate can contribute to lower peak pressures compared to diesel fuel, which typically lacks oxygen. The presence of additive could decline the level of peak pressures for all samples, while the enhancement of additive enhanced peak pressures. The α-MnO_2_/NCBs additive might affect oxygen availability and distribution during combustion. This can influence the combustion rate and the timing of peak pressures. The presence of oxygen in the additive can enhance the oxidation reactions and promote more complete combustion, leading to lower peak pressures. The peak pressures of SSGBs (second-generation) were around 86.58, 81.23, 76.04, 78, 79.98, 77.89, 78.89, 74.29, and 75.01 bar for SSGB5, SSGB10, SSGB20, SSGB5N50, SSGB5N100, SSGB10N50, SSGB10N100, SSGB20N50, and SSGB20N100, respectively. On the other hand, the values of peak pressures of GMCB were around 83.26, 78.21, 74.81, 74.61, 76.36, 72.61, 77.81, 69.66, and 72.31 for GMCB5, GMCB10, GMCB20, GMCB5N50, GMCB5N100, GMCB10N50, GMCB10N100, GMCB20N50, and GMCB20N100, respectively. SSGB5 had the maximum peak pressures among all samples, and it could be related to low CN and longer ignition delay, which follows the prior study.^36^ The higher peak pressures observed in SSGB5 compared with other samples can be attributed to a combination of factors. SSGB5 likely has a lower cetane number (CN), resulting in a longer ignition delay and slower combustion. The longer ignition delay allows for a larger amount of fuel to be present in the combustion chamber during compression, leading to higher peak pressures upon ignition. This, combined with the longer ignition delay itself, contributes to a more gradual and prolonged combustion process, further elevating the peak pressures.

In the absence of the additive, the high viscosity of SSGB5 can cause delays in the preparation of the air/fuel mixture, impacting the combustion process and resulting in high peak pressures. The high viscosity leads to poor fuel atomization and slower evaporation during fuel injection, causing a delay in fuel droplet vaporization and mixing with incoming air. This incomplete atomization and delayed fuel-air mixture preparation hinder efficient combustion, leading to delayed ignition and slower combustion rates. As a result, a larger amount of fuel remains in the combustion chamber during the compression stroke, contributing to an unexpected increase in in-cylinder pressure and ultimately higher peak pressures.^8^Figure 5 illustrates that the presence of an additive may improve ignition delay, which decreased viscosity and increased CN values and may affect the combustion stage and air–fuel mixture. Therefore, GMCB20N50 achieved the greatest reduction in peak pressures. There is a visible increase in viscosity with CCLs. As a result, GMCB20N50 presented lower viscosity and peak pressures results as compared to other samples. As a result of the increased quantity of double bonds, peak pressures also increase. The maximum unsaturated acids and double bonds were displayed via SSGB5 or second-generation groups, resulting in a great number of peak pressures. Thus, the fourth-generation fuel achieved the lowest peak pressure among all fuels.^37^

An additive was used to manipulate the rate of accumulated heat release (AHR) in biodiesels (Figure S2). Biodiesel AHR values increased in parallel with the TEMP and in-cylinder pressure. By enhancing biofuels in diesel, the value of AHR may also be enhanced; regarding Figure S2, the 20% biofuels may have better results than the lower double bonds gained via the GMCB group, which may affect the O/C and oxygen content, both of which affect AHR. It is noteworthy that FAs can enhance the value of CN because they can increase its value. CN advancement is influenced by the progress of CCLs. In contrast to the SSGB group, the superior CN group belongs to the GMCB group. The CN had a straight impact on HR, and the highest AHR was shown via the GMCB group.^38^ The adsorption of oxygen (O) on the surface of the additive and the release of oxygen from its lattice can significantly impact the combustion process. Oxygen adsorption probably provides an additional oxygen source, promoting a more complete fuel oxidation and leading to increased heat release.^39^ Furthermore, if the additive contains an oxygen lattice within its structure, then released oxygen reacts with the fuel, generating additional heat. Both the oxygen adsorption and oxygen release probably contribute to enhanced combustion and a higher accumulation of heat release.^40^ The *d* space, or lattice spacing, in the additive plays a significant role in influencing accumulated heat release during combustion. The *d* space probably affects the surface area of the additive with a smaller *d* space resulting in a larger surface area. This increased surface area provides more active sites for combustion reactions, enhancing fuel oxidation and facilitating higher heat release. Additionally, the *d* space influences the reactivity of the additive, with a smaller *d* space typically associated with increased reactivity. This heightened reactivity enables faster and more complete fuel combustion, contributing to a higher heat release. Moreover, the *d* space can impact the availability of oxygen within the additive’s lattice structure. A smaller *d* space allows for greater access to oxygen, facilitating its release during combustion.^41^ In the context of our comparative analysis, an essential parameter under scrutiny is the heat release rate (HRR), a pivotal factor that significantly distinguishes biodiesel formulations from their additives. The HRR represents the rate at which energy is liberated during combustion and is paramount in evaluating engine performance and emissions. Biodiesel, due to its intrinsic oxygen content stemming from ester groups, promotes a more comprehensive combustion process, resulting in a controlled and slower HRR when compared to that of conventional diesel fuel. This slower HRR leads to reduced peak pressures as energy release occurs over an extended duration. In contrast, the presence of specific additives, such as α-MnO_2_/NCBs, modulates oxygen availability during combustion, impacting the HRR. Additives capable of enhancing oxidation reactions foster a more complete combustion, translating to a lower HRR and subsequently decreased peak pressures. Additionally, the HRR is profoundly influenced by factors such as fuel viscosity, ignition delay, and fuel–air mixture preparation. For instance, elevated viscosity, as observed in SSGB5, can induce delays in air–fuel mixture preparation, resulting in incomplete combustion and higher peak pressures. Thus, a comprehensive understanding of HRR is essential for comparing the performance of various biodiesel formulations and additives, as it directly shapes engine efficiency and emission profiles.^14^

#### 3.2.2 Engine Torque and Power

**Figure 7:**
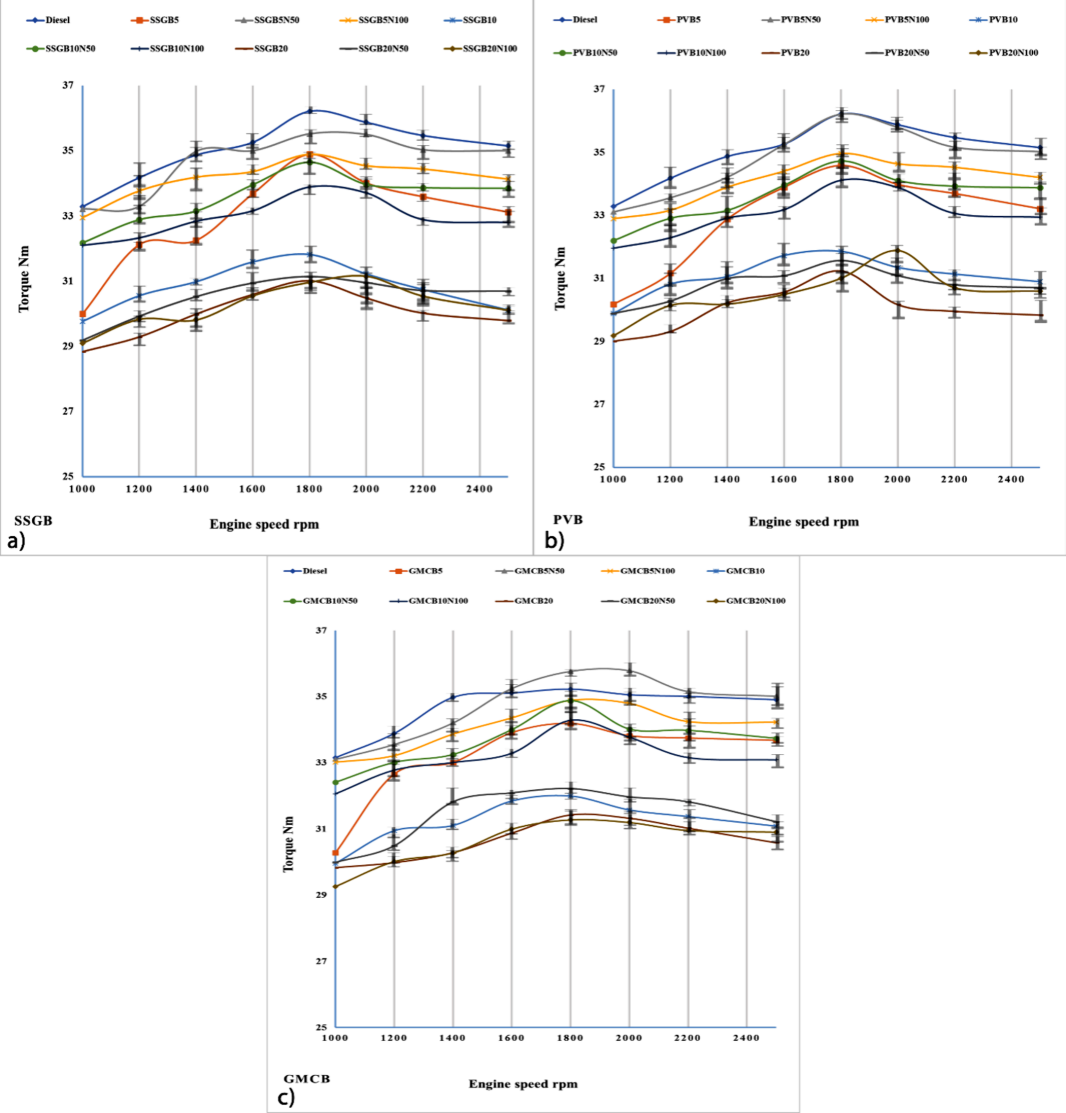
Level of torque for different levels of biodiesel and nanoadditive for (a) sweet-scented geranium biodiesel (SSGB), (b) *Pyropia vietnamensis* biodiesel (PVB), and (c) genetically modified canola biodiesel (GMCB).

Figure 7 presents torque measurements for different biodiesel generations. At 1800 rpm, the highest torque values were observed across all generations. Furthermore, the torque gradually decreased at this specific rpm for all samples. The incorporation of biofuels into diesel has generally resulted in a decrease in torque. However, the addition of additive at a concentration of approximately 50 ppm has been shown to enhance torque levels compared to pure biodiesel. At 1800 rpm, GMCB proved the maximum torque, and they were about 34.89, 35.77, 34.91, 32, 34.88, 34.29, 31.43, 32.22, and 31.28 Nm for GMCB5, GMCB5N50, GMCB5N100, GMCB10, GMCB10N50, GMCB10N100, GMCB20, GMCB20N50, and GMCB20N100, respectively. On the other hand, the minimum torque observed via the SSGB group was nearly 34.19, 35.52, 34.89, 31.82, 34.65, 33.89, 31.01, 31.14, and 30.96 Nm, respectively. The best results were obtained with 5% biodiesel in the presence of additive. Viscosity and HV are two factors that can influence the results, and the highest viscosity and HV were observed for 5% biodiesel with the additive. In addition to FAs and CCLs, viscosity can be influenced by CCLs, and GMCB5N50 had the longest CCLs of all samples, resulting in the highest viscosity.^42^ Compared with other samples, GMCB5N50 exhibited the highest viscosity and high HV. Saturated acids and CCLs can alter the parameter of HV, and GMCB5N50 exhibited the longest CCLs and highest saturated acids.^43^ Both factors could affect the power level for all biodiesels, and the highest power level was observed via GMCB5N50 (Figure S3). It was around 5.97, 6.43, 6.21, 5.31, 5.44, 5.35, 5.02, 5.26, and 5.18 kW (2500 rpm). The surface area of the additive plays a crucial role in enhancing combustion efficiency, thereby impacting the torque and power output. Increasing the surface area promotes better fuel oxidation and combustion, resulting in improved power output and higher torque.^40^ Additionally, the presence of an oxygen lattice within the additive’s structure acts as an oxygen reservoir, releasing oxygen during combustion. This released oxygen reacts with the fuel, contributing to increased heat release and subsequently higher gas pressures, leading to elevated torque and power.^44^ Furthermore, the adsorption of oxygen on the additive’s surface creates an oxygen-rich environment, promoting a more complete fuel combustion and increasing heat release. Moreover, the *d* space, or lattice spacing, of the additive affects reactivity and oxygen availability. A smaller *d* space correlates with increased reactivity and greater access to oxygen within the lattice, enhancing combustion efficiency and resulting in a higher heat release, torque, and power output.^45,46^

#### 3.2.3 Brake-Specific Fuel Consumption (BSFC)

**Figure 8:**
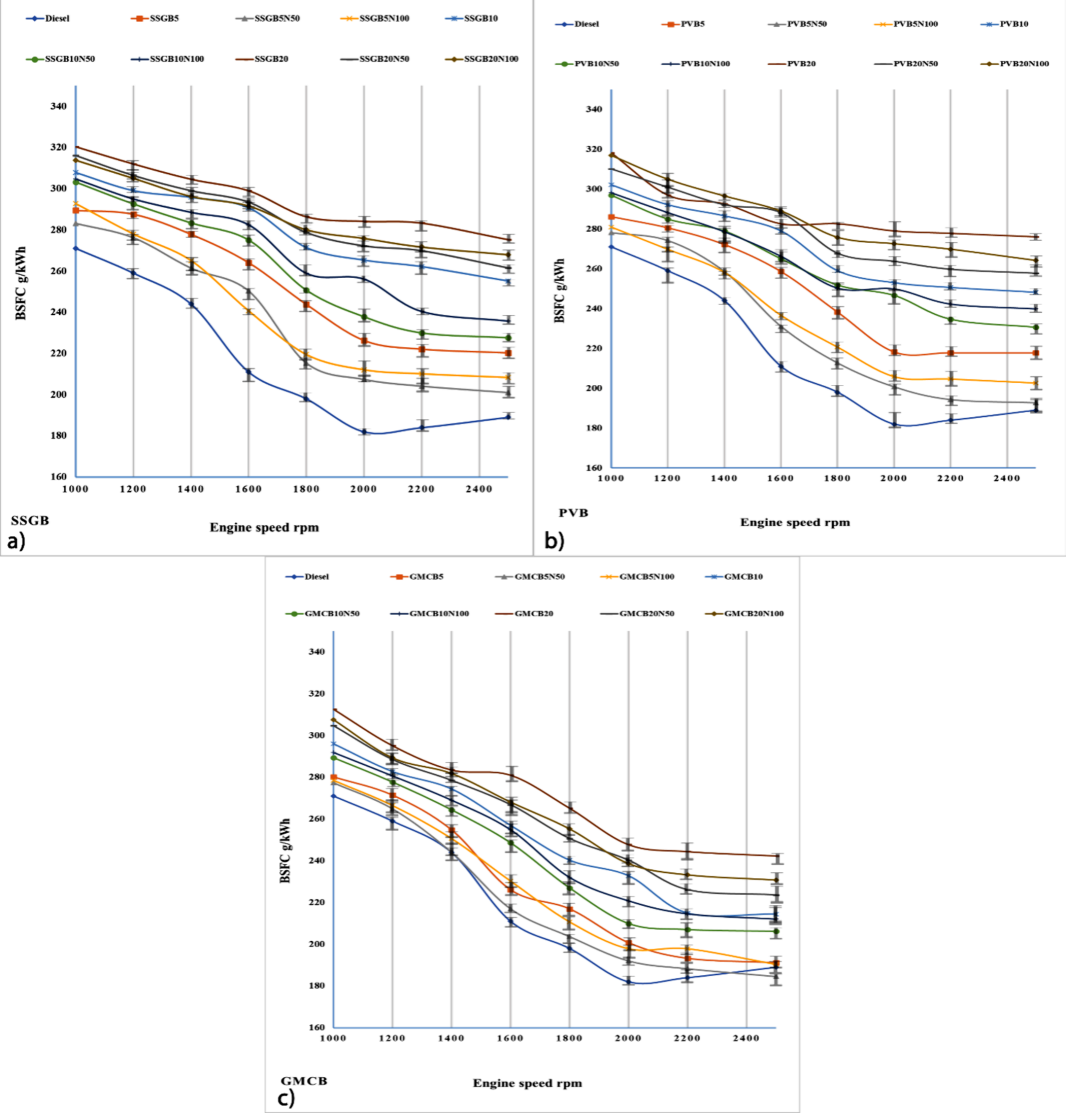
Level of BSFCs for different levels of biodiesel and nanoadditive for (a) sweet-scented geranium biodiesel (SSGB), (b) *Pyropia vietnamensis* biodiesel (PVB), and (c) genetically modified canola biodiesel (GMCB).

Figure 8 presents the BSFCs for each sample. As the engine speed decreases, there is a corresponding decrease in the BSFCs for all samples. However, between 2200 and 2400 rpm, a change in the rate of BSFC decline is observed, which can be attributed to the higher absolute fuel injection at higher engine speeds.^47^ The highest level of reduction was gained via the GMCB group compared to other samples, and they were around 32, 34, 33, 28, 29, 23, 27, and 26% for GMCB5, GMCB5N50, GMCB5N100, GMCB10, GMCB10N50, GMCB10N100, GMCB20, GMCB20N50, and GMCB20N100, respectively. Furthermore, the best outcomes were associated with fourth-generation families. The inclusion of additives resulted in larger reductions compared with using neat biodiesel without additives. This is likely attributed to the additives’ capacity to provide higher levels of oxygen for the combustion process, enhancing the overall combustion efficiency.^48^ There is a relationship between HV and viscosity, which can affect the reduction of BSFCs, and the decline and increase in viscosity and HV can also reduce BSFCs. The influence of FAs may be one of the factors contributing to the changes. Both viscosity and HV have been impacted by FAs, which may have a bearing on BSFC. A prolonged CCL can increase viscosity; however, GMCB had the highest values of O/C and saturated acids, and these factors are more likely to influence BSFCs. Enhanced additive can increase the level of BSFCs, and the greater level of biofuels in diesel can enhance BSFCs, which can decrease the HV and increase the viscosity of the fuel.^49^ The surface area of the additive probably plays a critical role in influencing BSFC by improving the fuel–air mixing and combustion efficiency. An increased surface area enhances combustion by facilitating better fuel–air mixing and reducing unburned fuel residues, resulting in improved BSFC values.^50^ Additionally, the presence of an oxygen lattice within the additive’s structure contributes to increased combustion efficiency. The release of oxygen from the lattice during combustion promotes a more complete fuel oxidation, reducing fuel wastage and leading to lower BSFC.^51^

#### 3.2.4 Brake Thermal Efficiency (BTE)

Figure S4 depicts the BTEs for different samples. The results reveal an inverse relationship between BTEs and BSFCs as well as between BTEs and HV. The addition of biofuels in diesel leads to an increase in BTEs, contrary to the trend observed for BSFCs. For instance, the highest level of BSFCs is observed for the 20% biofuel blend, while the highest level of BTEs is also observed for the 20% biofuel blend. The BTEs of biofuels are typically higher with or without additive due to the fact that the additives have a large surface-to-volume ratio, which demonstrates their high performance. There is a possibility that the presence of additive in samples will result in more effective blending between samples and air. The presence of oxygen in the additive and the high oxygen content in biofuels could enhance the high O/C ratio in the structure, leading to improved combustion. Comparing among all samples, GMCB20N50 had the supreme value of O/C, and it could be associated with high CCLs and saturated acids.^52^

#### 3.2.5 Exhaust Gas Temperature (EGT)

The exhaust gas temperature (EGT) is an important parameter that reflects the heat release during combustion (Figure S5). EGT values are influenced by various factors, including combustion duration, heat release rate, and other parameters. In this study, a clear inverse relationship between BTEs and EGTs can be observed. As engine speeds increased, all samples exhibited higher EGT values, indicating an enhancement in heat release during combustion. The EGTs for the diesel fuel were consistently lower than those for all other samples across all engine speeds. As the biodiesel content increased, the EGTs also showed an increase, indicating a higher level of heat release during combustion. The minimum EGT values were observed via GMCB at 1000 rpm, and they were about 269, 290, 297, 307, 304, 311, 323, 322, and 330 °C for GMCB5, GMCB5N50, GMCB5N100, GMCB10, GMCB10N50, GMCB10N100, GMCB20, GMCB20N50, and GMCB20N100, respectively. At 2500 rpm, the values were about 363, 356, 363, 372, 370, 380, 394, 389, and 395 °C. The highest EGTs (at 1000 rpm) were about 299, 294, 296, 312, 310, 315, 332, 327, and 338 °C, and at 2500 rpm, they were around 375, 368, 376, 396, 388, 395, 412, 406, and 414 °C. It is true that biofuels in diesel have a lower HV, which influences the level of EGTs, and the use of additive could improve the outcomes of EGTs. CN is considered to be a parameter that influences the value of EGTs. Low CN results in a longer ignition delay, resulting in high EGTs and deferred combustion. CN amounts can be manipulated via saturated acids and CCLs. The increase in unsaturated acids may result in a decrease in the level of CN, but the relationship between CN and CCLs is not linear. Consequently, the presence of additive in the compound may promote the value of CN, saturated acids, and longer CCLs, which all impact the quantity of emissions.^53^ The surface area, oxygen lattice, oxygen adsorption, and *d* space of the additive can indirectly impact the relationship between BTE and exhaust gas temperatures (EGTs). Increasing the surface area enhances combustion efficiency and reduces heat loss, leading to lower EGTs and improved thermal efficiency, contributing to higher BTEs.^54,55^ The presence of an oxygen lattice and the adsorption of oxygen on the additive’s surface enhance combustion efficiency, reducing waste heat and unburned fuel, which indirectly lowers EGTs and supports higher BTEs. The *d* space influences reactivity and oxygen availability, affecting combustion efficiency and waste heat generation. A smaller *d* space often correlates with improved combustion efficiency, resulting in lower EGTs and higher BTEs.

#### 3.2.6 CO_2_ Emission

**Figure 9:**
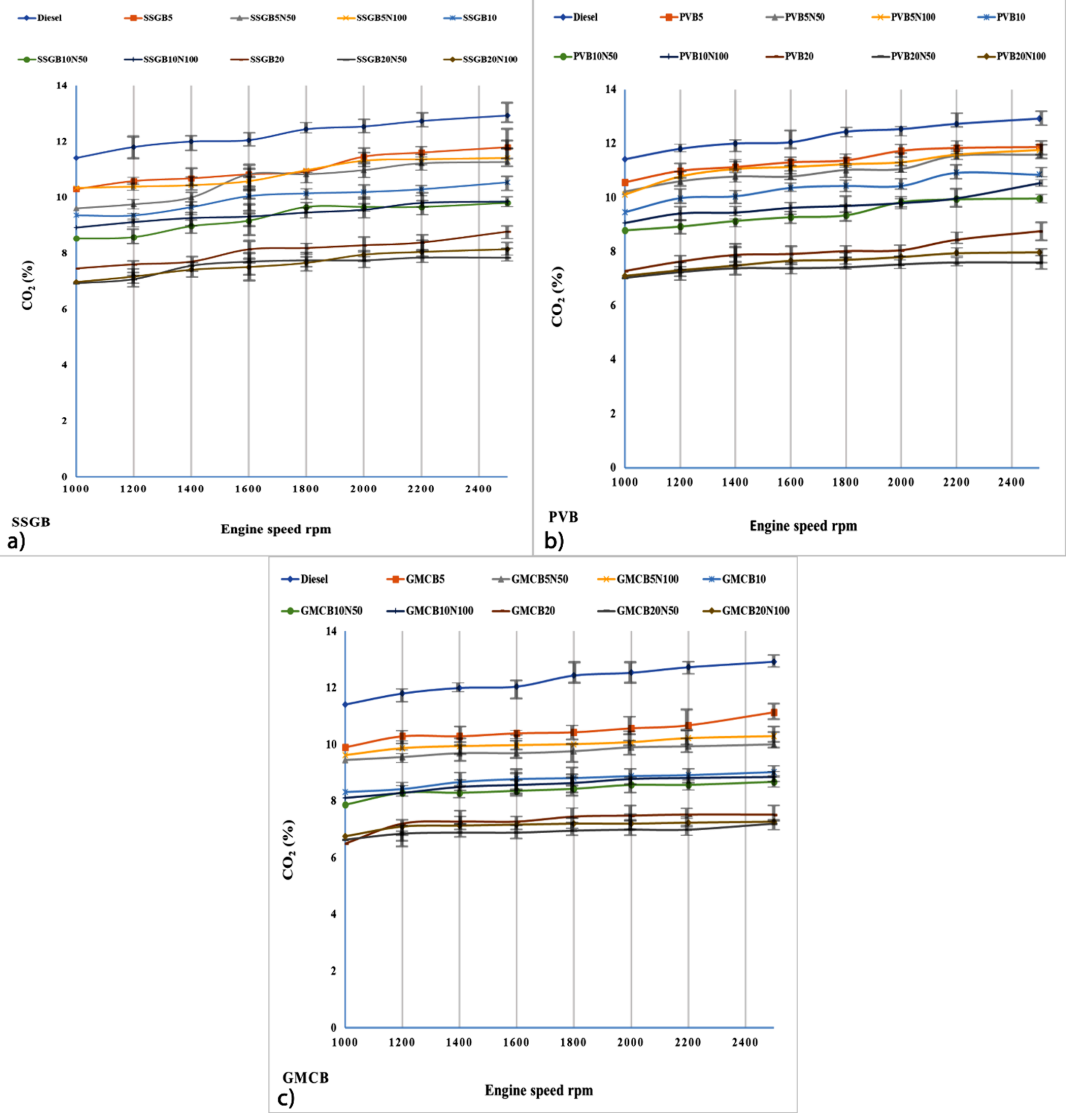
Level of CO_2_ for different levels of biodiesel and nanoadditive for (a) sweet-scented geranium biodiesel (SSGB), (b) *Pyropia vietnamensis* biodiesel (PVB), and (c) genetically modified canola biodiesel (GMCB).

Figure 9 depicts the CO_2_ emission levels observed in different samples. The incorporation of biofuel into diesel led to a reduction in the level of CO_2_ emissions. Notably, the fourth generation of biodiesel exhibited lower CO_2_ levels compared to those of other generations, both with and without additives. The GMCB group presented levels of CO_2_ around 9.9, 9.45, 9.65, 8.33, 7.87, 8.12, 6.51, 6.65, and 6.75% for GMCB5, GMCB5N50, GMCB5N100, GMCB10, GMCB10N50, GMCB10N100, GMCB20, GMCB20N50, and GMCB20N100 (at 1000 rpm). The values of CO_2_ were observed about 11.13, 10.01, 10.29, 9.03, 8.68, 8.85, 7.52, 7.21, and 7.28% for GMCB5, GMCB5N50, GMCB5N100, GMCB10, GMCB10N50, GMCB10N100, GMCB20, GMCB20N50, and GMCB20N100 (at 2500 rpm). The results were against the former report, which claimed that the presence of biofuels could enhance the value of CO_2_.^56^ However, the findings of this study contradicted the hypothesis of complete combustion. Surprisingly, the fourth generation exhibited higher O/C and H/C ratios compared to previous generations, and the incorporation of additives had an impact on these elemental compositions, leading to lower CO_2_ emissions. The substantial O/C and H/C ratios were attributed to CCLs and saturated acids. Notably, the GMCB group with NCBs demonstrated a superior performance in reducing CO_2_ emissions.

The surface area, oxygen lattice, oxygen adsorption, and *d* space of the additive have the potential to influence the CO_2_ reduction in combustion processes. Increasing the surface area enhances fuel–air mixing and combustion efficiency, leading to reduced formation of CO and incomplete combustion products.^57^ The presence of an oxygen lattice promotes more complete fuel oxidation, indirectly supporting CO_2_ reduction by minimizing the amount of CO emissions. Oxygen adsorption on the additive’s surface creates an oxygen-rich environment, facilitating efficient fuel combustion and reducing CO formation, thereby contributing to CO_2_ reduction.^45,58^ The *d* space affects reactivity and oxygen availability, improving combustion and further aiding in the CO_2_ reduction.

#### 3.2.7 CO Emission

Figure S6 illustrates the CO emissions for different fuel blends. It can be observed that all samples exhibited a decrease in CO emissions as the engine speeds increased. Particularly, the fourth generation of biodiesels showed the most significant reduction in CO emissions compared to the other fuel samples. The values of GMCB were around 640, 596, 615, 453, 415, 419, 285, 223, and 257 ppm for GMCB5, GMCB5N50, GMCB5N100, GMCB10, GMCB10N50, GMCB10N100, GMCB20, GMCB20N50, and GMCB20N100, respectively (at 1000 rpm). This reduction at 2500 rpm was around 416, 369, 397, 288, 251, 279, 220, 195, and 204 ppm. The SSGB group exhibited the highest level of CO emissions among all samples. However, the addition of 20% biofuels with the additive demonstrated the best performance in reducing CO emissions. The decrease in the CO emissions in the cylinder can be attributed to the presence of oxygen, which facilitates a more efficient combustion at high temperatures. This is supported by the higher oxygen content in the long carbon chain lengths, resulting in cleaner and more complete combustion. Moreover, methyl esters with longer CCLs possess higher boiling and melting points, making them less volatile and more prone to incomplete combustion, thereby increasing the level of CO emissions. The GMCB group, which exhibited longer CCLs and higher O/C ratios, demonstrated a contrasting relationship between CO emissions and O/C content.^59^

As a result, the second generation of biodiesel exhibited the highest CO emissions compared with the other biodiesel generations. The increased viscosity of the fuel at engine speeds affected the atomization process, leading to challenges in achieving optimal air–fuel mixing. This, in turn, necessitated the introduction of additional air during the combustion process.^60^

#### 3.2.8 HC Emission

**Figure 10:**
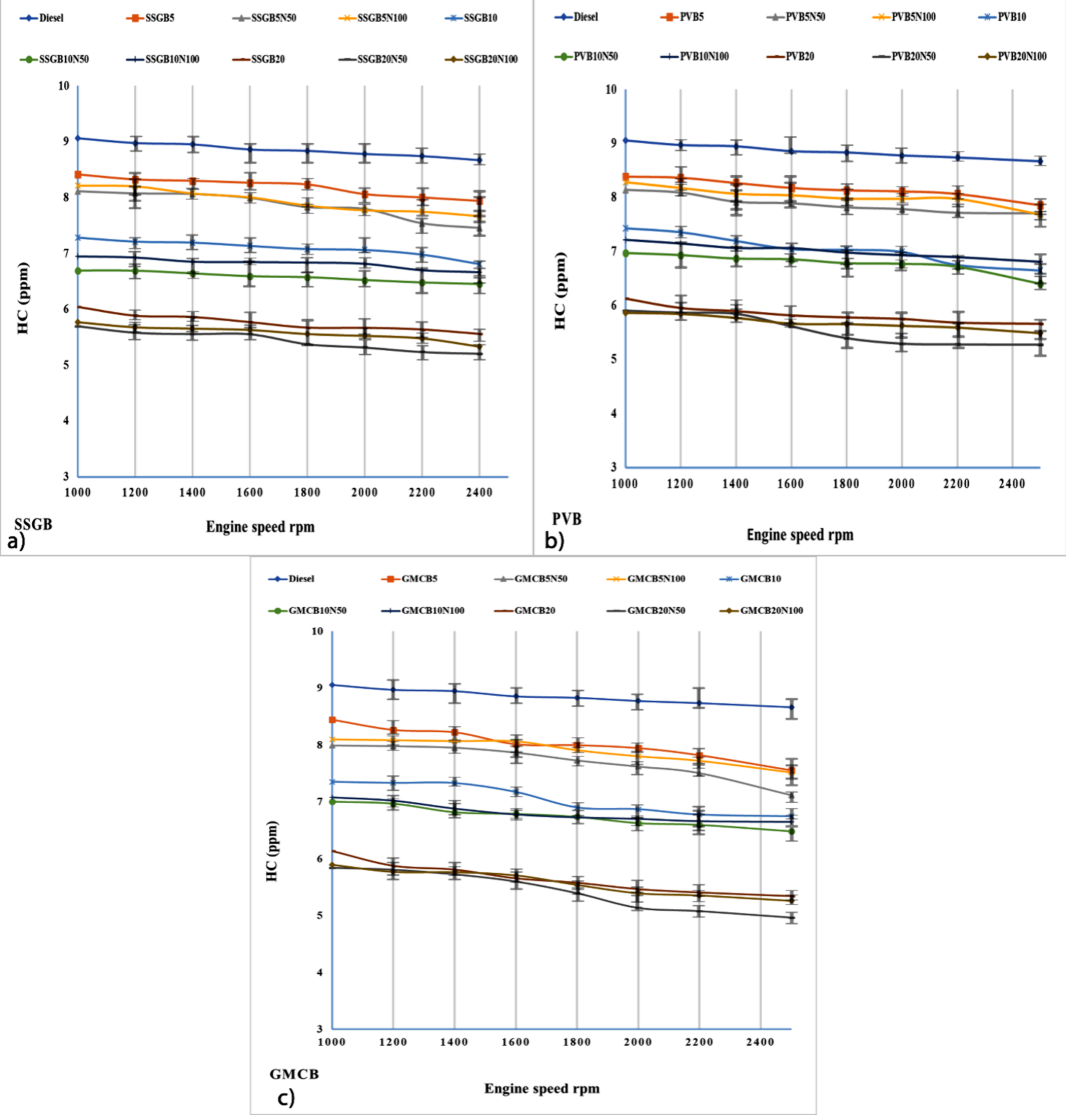
Level of HC for different levels of biodiesel and nanoadditive for (a) sweet-scented geranium biodiesel (SSGB), (b) *Pyropia vietnamensis* biodiesel (PVB), and (c) genetically modified canola biodiesel (GMCB).

Figure 10 displays the HC emissions for different fuel samples. HC emissions are associated with incomplete combustion. The incorporation of biofuels into diesel led to a decrease in HC levels, and this reduction could be attributed to the presence of α-MnO_2_/NCBs as additives in the fuel blend. The lowest value of HC was observed via GMCBs (fourth-generation) compared with other samples. The values are around 8.44, 7.98, 8.09, 7.35, 7, 7.08, 6.13, 5.84, and 5.98 ppm for GMCB5, GMCB5N50, GMCB5N100, GMCB10, GMCB10N50, GMCB10N100, GMCB20, GMCB20N50, and GMCB20N100, respectively (at 1000 rpm). However, the values declined at 2500 rpm, which were around 7.55, 7.11, 7.52, 6.74, 6.48, 6.65, 5.34, 4.96, and 5.26 ppm for GMCB5, GMCB5N50, GMCB5N100, GMCB10, GMCB10N50, GMCB10N100, GMCB20, GMCB20N50, and GMCB20N100, respectively. Under high load conditions, incomplete combustion may occur when excess fuel is injected into the engine. However, the presence of biofuels, which contain a significant amount of oxygen, can promote complete combustion. Among the different biodiesel types, GMCB exhibited the highest O/C ratio, leading to the greatest reduction in HC emissions. The findings suggest that the additive used in the study may influence the O/C ratio, thereby enhancing the combustion efficiency and reducing HC emissions. Furthermore, the enhancement in the exhaust gas temperature and CN for biodiesels can be attributed to certain factors. Higher exhaust gas temperatures can prevent the condensation of heavier hydrocarbons, thereby reducing the level of formation of particulate matter. Additionally, the superior CN of biodiesels results in shorter combustion times, leading to a more complete combustion and a decrease in HC emissions.^61^ The influence of FAs on CN has been established, and it has been observed that an increase in the level of saturated acids and CCLs can lead to higher CN values. In the case of GMCB, which possesses longer saturated acids and CCLs, it exhibited lower maximum HC emissions compared with other samples. However, the prolonged CCLs can promote HC levels due to their higher boiling point, resulting in a decrease in the O/C ratio and an insufficient oxygen supply for complete combustion, leading to the accumulation of HC emissions.^2^

The surface area, oxygen lattice, oxygen adsorption, and *d* space of the additive have the potential to influence the reduction of HC emissions in combustion processes. Increasing the surface area enhances fuel–air mixing and combustion efficiency, leading to a more complete fuel oxidation and reduced formation of unburned hydrocarbons, thereby contributing to HC reduction.^62^ The presence of an oxygen lattice within the additive promotes improved combustion efficiency and facilitates more complete fuel oxidation, minimizing the formation of unburned hydrocarbons.^63^ Oxygen adsorption on the additive’s surface creates an oxygen-rich environment, enhancing fuel combustion and reducing the production of unburned hydrocarbons, supporting HC reduction.^45^ The *d* space affects reactivity and oxygen availability, promoting a more complete fuel oxidation and reducing the formation of unburned hydrocarbons.

#### 3.2.9 Soot Emission

**Figure 11:**
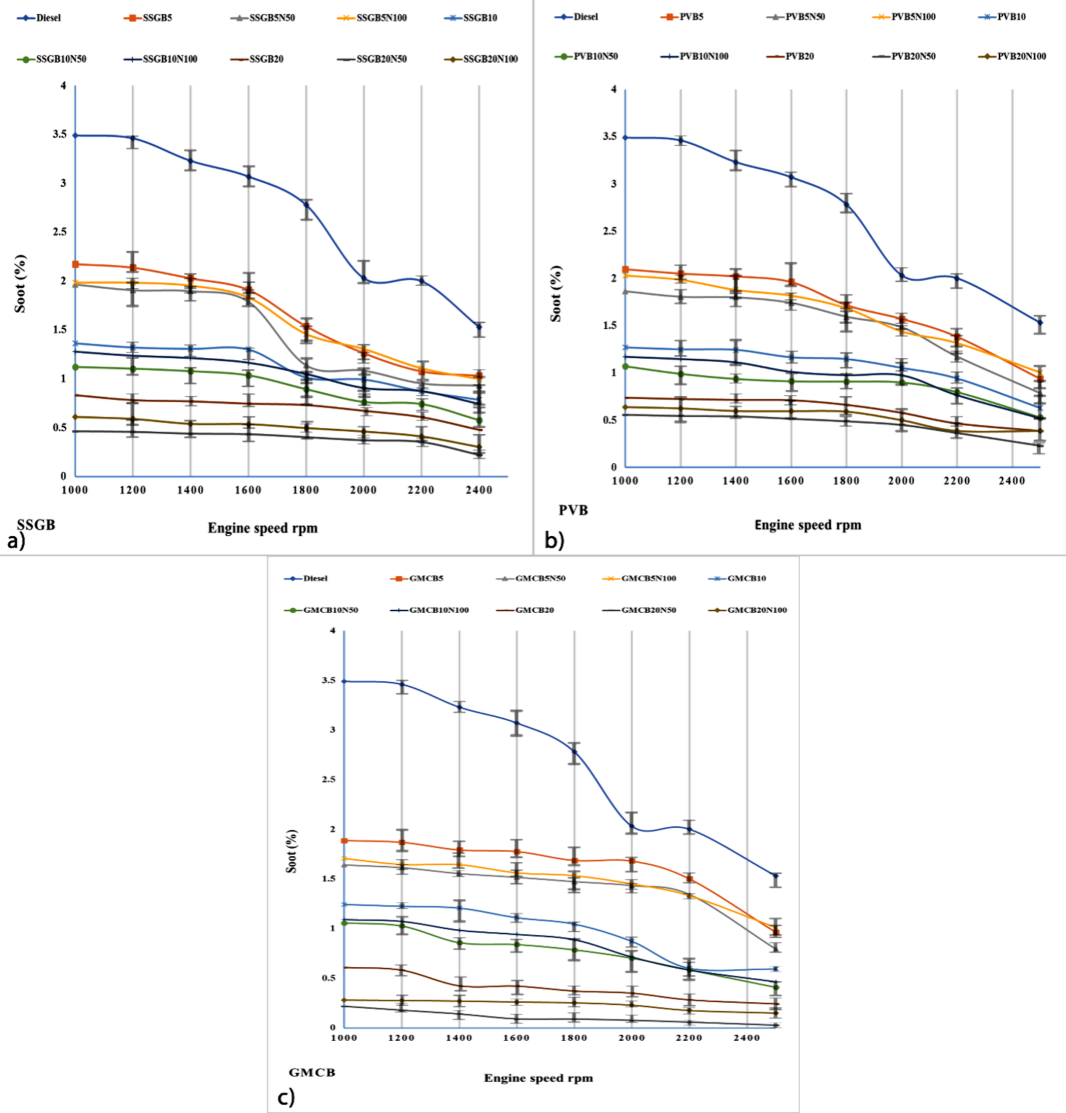
Level of soot values for different levels of biodiesel and nanoadditive for (a) sweet-scented geranium biodiesel (SSGB), (b) *Pyropia vietnamensis* biodiesel (PVB), and (c) genetically modified canola biodiesel (GMCB).

Figure 11 displays the particulate matter (soot) emissions for different fuel samples. The addition of biofuels to diesel resulted in a decrease in soot emissions for all samples, and the presence of NCBs further enhanced this reduction. Among all the samples, GMCB or the fourth-generation biodiesel exhibited the greatest reduction in soot emissions compared to other samples. The lowest soot values for the GMCB group were about 1.88, 1.63, 1.70, 1.24, 1.05, 1.08, 0.60, 0.21, and 0.27 vol% for GMCB5, GMCB5N50, GMCB5N100, GMCB10, GMCB10N50, GMCB10N100, GMCB20, GMCB20N50, and GMCB20N100, respectively (at 1000 rpm); however, this reduction was round 0.96, 0.79, 1, 0.59, 0.40, 0.46, 0.24, 0.02, and 0.14 vol% for GMCB5, GMCB5N50, GMCB5N100, GMCB10, GMCB10N50, GMCB10N100, GMCB20, GMCB20N50, and GMCB20N100, respectively (at 2500 rpm). Based on the findings regarding soot emissions, the increase in CCL was found to have an impact on reducing soot values. This contradicts the findings of Jafarihaghighi et al.’s report, which suggested that CCL enhancement does not affect oxygen content. However, it is worth noting that the extension of CCL can contribute to higher O/C ratios and increased oxygen content, which can result in a reduction in soot emissions.^5^ Results proved that the additive could promote the oxygen content, and the GMCB20N50 sample verified the most significant soot value reduction. The SSGB group exhibited higher levels of unsaturated acids and double bonds compared with the GMCB group. This contrast in unsaturated acid content and the presence of a double bond had a direct relationship with the soot values. In other words, an increase in unsaturated acids and double bonds corresponded to higher soot emissions.^64^ The H/C ratio and saturated acid values could also decrease soot emissions, and the GMCB group had the highest H/C and saturated acid values, resulting in lower soot levels. Surface area, oxygen lattice, oxygen adsorption, and d space of the additive have the potential to significantly impact the reduction of soot emissions in combustion processes. By increasing the surface area, the additive promotes better fuel-air mixing and combustion efficiency, facilitating more thorough fuel oxidation and reducing the formation of soot particles.^65,66^ A larger surface area enables improved contact between the fuel and additive, enhancing the combustion process and mitigating the generation of soot. Furthermore, the presence of an oxygen lattice within the additive’s structure plays a crucial role in enhancing combustion efficiency. The lattice acts as an oxygen reservoir, providing additional oxygen during combustion. This increased oxygen availability aids in the complete oxidation of fuel molecules, minimizing the formation of soot particles.^67,68^ By ensuring sufficient oxygen for the combustion process, the oxygen lattice helps to prevent incomplete combustion that leads to soot formation. Additionally, the adsorption of oxygen on the additive’s surface creates an oxygen-rich environment, further enhancing fuel combustion. This oxygen-rich environment facilitates the complete oxidation of fuel molecules, reducing the presence of partially oxidized compounds that can contribute to soot formation. The process of oxygen adsorption on the additive’s surface promotes more efficient combustion, thereby reducing soot emissions.^69,70^ The d space or lattice spacing of the additive also influences the combustion process. A smaller d space typically correlates with increased reactivity and improved oxygen access. This facilitates more complete fuel oxidation and reduces the formation of soot particles.^45,71^

#### 3.2.10 NOx Emission

**Figure 12:**
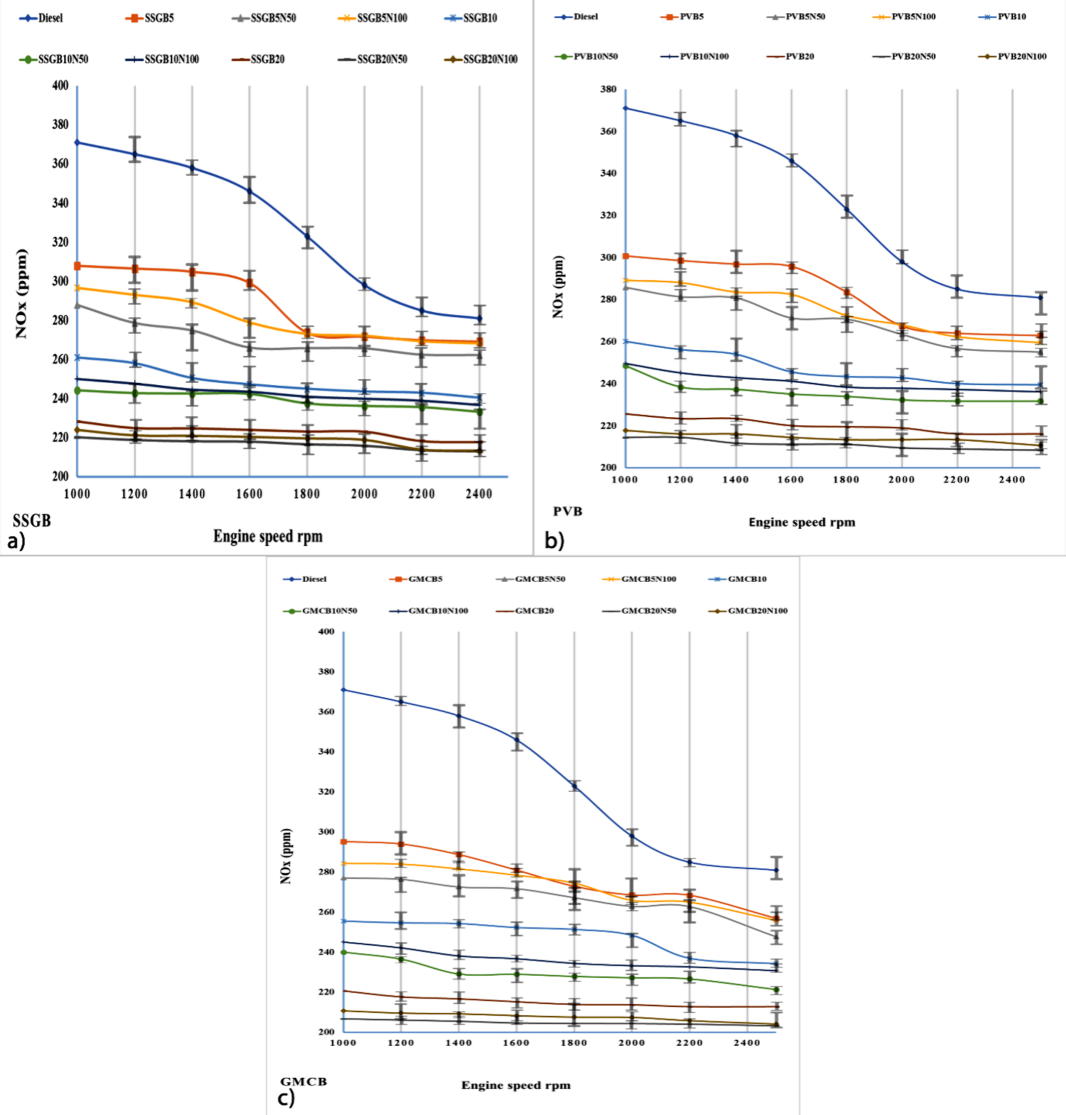
Level of NOx values for different levels of biodiesel and nanoadditives for (a) sweet-scented geranium biodiesel (SSGB), (b) *Pyropia vietnamensis* biodiesel (PVB), and (c) genetically modified canola biodiesel (GMCB).

Figure 12 depicts the levels of NOx emissions for different samples. Consistent with previous research, the highest NOx values were observed under maximum load conditions. This increase can be attributed to the higher fuel injection rates, which lead to elevated flame temperatures. The higher flame temperature promotes the formation of thermal NOx. Additionally, the higher flame temperature allows for greater oxygen dissociation, providing oxygen atoms that can combine with nitrogen to form NOx and nitrogen oxides. The incorporation of additive in the samples resulted in lower NOx values compared to other samples without the additive. This can be attributed to the additive’s ability to enhance convective heat transfer. As a result, the in-cylinder temperature is reduced, leading to a decrease in thermal NOx formation. The reduction in temperature is facilitated by the interaction between the nanoparticles and water vapor present in the samples, which promotes the formation of highly reactive hydroxyl radicals. Additionally, the additive acts as a heat sink within the combustion chamber, effectively reducing the temperature and preventing the formation of hot zones that contribute to NOx generation. These findings are consistent with previous reports.^72^ The GMCB group presented minimum levels of NOx among all groups, and at 1000 rpm they were around 295, 277, 284, 255, 239, 245, 220, 206, and 210 ppm for GMCB5, GMCB5N50, GMCB5N100, GMCB10, GMCB10N50, GMCB10N100, GMCB20, GMCB20N50, and GMCB20N100, respectively. At 2500 rpm, the values were nearly 256, 247, 255, 234, 221, 230, 212, 203, and 204 ppm for GMCB5, GMCB5N50, GMCB5N100, GMCB10, GMCB10N50, GMCB10N100, GMCB20, GMCB20N50, and GMCB20N100, respectively. The CN values have an influence on the ignition characteristics, where higher CN values indicate a shorter ignition delay, resulting in lower fuel energy in premixed stages and decreased NOx emissions during these stages. Among all samples, the GMCB group exhibited the highest CN value, and increasing the proportion of biodiesel led to an increase in CN. The amount of NOx is associated with combustion duration, volumetric efficiency, and the temperature rise caused by the high activation energy required for the reactions. Due to the increase in volumetric efficiency and improved gas flow dynamics within the engine, the elevated EGT had an opposite effect on NOx emissions. This is attributed to the enhanced mixing of fuel and air, leading to a reduction in ignition delay.^4,49,73^ The increase in the concentration of unsaturated acids was found to have a negative impact on NOx emissions, and GMCB exhibited the lowest levels of unsaturated acids among the tested samples, which aligns with previous studies but contradicts the findings of Schonborn et al.^74^ Some outcomes implied that the attendance of oxygen could boost the value of NOx emissions,^75^ and the influence of O/C had affect the NOx value, which agreed with former study.^64^

Increasing the concentration of saturated acids and reducing the number of double bonds leads to an improvement in the CN and a decrease in the level of NOx.^64^

## 4 Conclusions

In this study, the use of α-MnO_2_/NCBs as an additive in biodiesel has been explored for the purpose of improving engine performance and reducing pollution. The investigation focused on second-, third-, and fourth-generation bacteria, which have demonstrated tolerance to adverse weather conditions and pose no threat to the human food chain. The fourth generation has been genetically modified to be unsuitable for human consumption, thus mitigating any potential impact on the food chain. The findings of this research highlight the direct influence of α-MnO_2_/NCBs on engine performance and the environment. Through a comparative analysis of all generations, it has been established that the nanoadditive can significantly enhance the performance of biodiesel. Notably, the fourth generation with α-MnO_2_/NCBs exhibited superior efficiency and demonstrated considerable growth potential. The study reveals that a dosage of approximately 50 ppm with a 5% biodiesel blend yielded optimal results for the nanoadditive. Based on the data presented, it is evident that the fourth-generation nanoadditive outperformed diesel fuel in terms of both efficiency and pollution control in various scenarios. Furthermore, the impact of specific factors related to the nanoadditive, such as the presence of an oxygen lattice, oxygen adsorption, *d* space (lattice spacing), and surface area, have been investigated in this research. The presence of an oxygen lattice within the additive’s structure has been found to enhance combustion efficiency and contribute to more complete fuel oxidation, thereby reducing pollutant emissions, including soot and hydrocarbons. Oxygen adsorption on the additive’s surface creates an oxygen-rich environment, further promoting efficient combustion and reducing emissions. The optimization of the *d* space influences the reactivity and oxygen availability within the additive, facilitating improved combustion and pollutant reduction. Moreover, the increased surface area of the additive enhances fuel–air mixing and combustion efficiency, resulting in reduced emissions. The effects of these factors on engine performance and pollutant reduction have been systematically evaluated in the conducted tests, providing valuable insights into the mechanisms underlying the improved performance and pollution control achieved by the nanoadditive.

## Supporting Information Available

Synthesis of α-MnO_2_/NCBs; (Table S1) properties of SSGB; (Table S2) properties of PVB; (Table S3) properties of GMCB; (Table S4) results of XPS for the additive; (Table S5) evaluating fatty acids; (Figure S1) schematic diagram for the biodiesel production and engine test; (Figure S2) accumulated heat value for all samples; (Figure S3) level of power for all samples; (Figure S4) level of BTEs for all samples; (Figure S5) level of EGTs for all samples; (Figure S6) level of CO for all samples (PDF)

## Nomenclature

AHR: accumulated heat release
BP: brake power
BSFC: brake-specific fuel consumption
CCLs: carbon chain lengths
CO: carbon monoxide
CNTs: carbon nanotubes
CN: cetane number
EGT: exhaust gas temperature
EGTs: exhaust gas temperatures
FA: fatty acid
GC: gas chromatography
GMCB: genetically modified canola biodiesel
HRR: heat release rate
HV: heating value
HRTEM: high-resolution transmission electron microscopy
HC: hydrocarbons
MWCNTs: multiwalled carbon nanotubes
NPs: nanoparticles
NAs: nanoadditives
NCB: nanocarbon ball
NOx: nitrogen oxides
O/C and H/C: oxygen-to-carbon ratio and hydrogen-to-carbon ratio
PVB: *Pyropia vietnamensis* biodiesel
SSGBl: sweet-scented geranium biodiesel
BTE: brake thermal efficiency
TiO_2_: titanium dioxide
UHCs: unburned hydrocarbons
WEB: water-emulsified biodiesel
XRD: X-ray diffraction
