# Supplementary material for: Innovative α-MnO2/Nanocarbon Ball Additive for Enhancing the Molecular Structure, Emission Control, and Engine Performance of Diverse Biodiesel Generations
Source: ACS Omega. 2024 Jan 25;9(5):5278–97. doi: 10.1021/acsomega.3c05848 (PMC10851363; doi:10.1021/acsomega.3c05848)
Supplement: Supplementary file 1 — ao3c05848_si_001.pdf [file ao3c05848_si_001.pdf]

Supporting Information

Innovative α-MnO_2/nano Carbon Balls Additive for Enhancing Molecular Structure, Emissions Control, and Engine Performance of Diverse Biodiesel Generations

Ahmad Yousefvand[a], Mehdi Ardjmand[a,b*], Hamidreza Mogadamzadeh[a]

[a.]Department of Chemical Engineering, South Tehran Branch, Islamic Azad University, Tehran, Iran
[b.]Nanotechnology Research Center, Tehran South Branch, Islamic Azad University, Tehran, Iran

Synthesis of α-MnO_2/NCBs

Carbon Nano Balls were made by incomplete combustion at 800 ˚C using oil residue as a feedstock. It was conducted in a vertical reactor with a residence time of less than one minute. All stages of the reaction were supposed to be carried out at a flow rate of 200 cc/min of nitrogen gas.

After the nano additive and biodiesel have been synthesized and the quality of the production of the biodiesel and nano additive has been confirmed, fuel samples are collected for testing, combining nano additive with two concentrations between 50 and 100 ppm. Further, these two concentrations were selected because it is generally not recommended to use concentrations exceeding 100 ppm for environmental reasons.

A 30-min ultrasonic bath at 303 k and 40 Hz was used to ensure proper distribution and stability of the samples. Since ultrasonics cause less destruction of carbon nanoballs than other methods of distribution, ultrasonics are preferred.

A homogeneous solution was created by combining 0.525g $MnSO_4.H_2O$ and 1.25g $KMnO_4$ in 80ml of distilled water, followed by the addition of 2 ml of 68% nitric acid and magnetic stirring for approximately one hour. The resulting solution was transferred into a Teflon stainless steel autoclave with a volume of 50 ml. The autoclave was subjected to a 28-hour oven cycle at 100 °C, followed by cooling to room temperature. The resulting product was then concentrated, centrifuged, and dried for 14 hours at 60°C to obtain α-MnO_2. For the preparation of α-MnO_2/NCBs, the same procedure was followed, with the only difference being the use of a 100 ml autoclave and the addition of supports during the synthesis of α-MnO_2.

The study employed various analytical techniques to investigate the characteristics of the samples. Powder X-ray diffraction (PXRD) was performed using a Brucker D8 Advance diffractometer with Cu Kα radiation, operating at specific voltage and current settings. The diffraction pattern was collected within a defined range of 2θ angles to determine the crystal structures of the different materials. transmission electron microscopy (TEM) was conducted using Hitachi instruments. Here is an image of a carbon nano-ball that has a spherical shape and is approximately 70 nanometers in diameter. TEM techniques were used to identify the samples. To determine the morphology of the samples, the Materials and Energy Research Institute used a scanning electron microscope (TEM) model CM-200FEG (200 kV) manufactured by Philips (Austria). The low-temperature nitrogen adsorption-desorption method was utilized to assess the special surface area and pore volume, employing a Micromeritics ASAP 2020 instrument. The samples were subjected to specific heating and vacuum conditions before adsorption to ensure accurate measurements. X-ray photoelectron spectroscopy (XPS) was employed to determine the oxidation states, using a Kratos AXIS Supra system equipped with specific components and operating under ultra-high vacuum (UHV) conditions. The system allowed for the collection of survey scan spectra and high-resolution scans of selected regions with precise energy steps, pass energy, accelerating voltage, and emission current settings.

Table S1. Properties of SSGB in various blends.

Fuel	Cetane Number (CN)	Heating Value kJ/kg (HV)	Viscosity (mm^2/s (40˚C))
ASTM test	D613	D2015	D445
Diesel	47	44880	2.59
SSGB5	51	43980	2.93
SSGB5N50	56	44410	2.68
SSGB5N100	54	44100	2.88
SSGB10	50	43910	2.97
SSGB10N50	55	44010	2.67
SSGB10N100	54	44000	2.70

SSGB20	50	43870	3.21
SSGB20N50	54	44010	2.83
SSGB20N100	52	43990	2.84

Table S2. Properties of PVB in various blends.

Fuel	Cetane Number	Heating Value kJ/kg	Viscosity (mm^2/s (40˚C))
ASTM test	D613	D2015	D445
Diesel	47	44880	2.59
PVB5	53	44010	2.90
PVB5N50	58	44550	2.67
PVB5N100	57	44400	2.69
PVB10	51	43990	2.93
PVB10N50	57	44390	2.71
PVB10N100	56	44380	2.72
PVB20	50	43890	3.01
PVB20N50	55	44350	2.73
PVB20N100	54	44330	2.75

Table S3. Properties of GMCB in various blends.

Fuel	Cetane Number	Heating Value kJ/kg	Viscosity (mm^2/s (40˚C))
ASTM test	D613	D2015	D445
Diesel	47	44880	2.59
GMCB5	54	44420	2.91
GMCB5N50	61	44690	2.60
GMCB5N100	59	44500	2.62
GMCB10	55	44410	2.95
GMCB10N50	59	44580	2.63

GMCB10N100	57	44490	2.65
GMCB20	56	44100	3.09
GMCB20N50	58	44470	2.67
GMCB20N100	57	44450	2.68

Table S4. the results of XPS for additive

Catalyst	Peak (eV)	Area%	$O_{latt\%}$	$O_{ads\%}$	$O_{latt\%}/O_{ads\%}$
α-MnO_2	Mn^{3+} 641.87<br>Mn^{4+} 643.07<br>$O_{latt\%}$ 530.62<br>$O_{ads\%}$ 531.80	Mn^{3+} 64.33<br>Mn^{4+} 35.67<br>Mn^{3+} / Mn^{4+} 1.8	60.29	39.71	1.59
α-MnO_2/NCBs	Mn^{2+} 639.85<br>Mn^{3+} 641.90<br>Mn^{4+} 644.01<br>$O_{latt\%}$ 528.71<br>$O_{ads\%}$ 529.90<br>$O_{surf\%}$ 532.38	Mn^{2+} 56.09<br>Mn^{3+} 35.93<br>Mn^{4+} 7.96<br>Mn^{2+} / Mn^{3+} 1.56<br>Mn^{3+} / Mn^{4+} 4.51	50.01	49.99	1.00

Table S5. Evaluating FAs in second, third, and fourth generation's samples

Fatty acid	SSGB % Wt	PVB % Wt	GMCB % Wt
Caprylic, C8:0 ($C_8H_{16}O_2$)	-	-	-
Capric, C10:0 ($C_{10}H_{20}O_2$)	-	1.10	-
Lauric, C12:0 ($C_{12}H_{24}O_2$)	-	-	2.00
Myristic, C14:0 ($C_{14}H_{28}O_2$)	-	-	-
Myristoleic, C14:1 ($C_{14}H_{26}O_2$)	-	3.36	-
Pentadecanoic, C15:0 ($C_{15}H_{30}O_2$)	-	3.02	-
Palmitic, C16:0 ($C_{16}H_{32}O_2$)	21.29	28.58	39.75
Palmitoleic, C16:1 ($C_{16}H_{30}O_2$)	1.00	0.16	-
Heptadecanoate, C17:0 ($C_{17}H_{33}O_2$)	-	1.48	-
Stearic, C18:0 ($C_{18}H_{36}O_2$)	2.40	4.00	5.30

Oleic, C18:1 ($C_{18}H_{34}O_2$)	52.05	18.02	23.65
Linoleic, C18:2 ($C_{18}H_{32}O_2$)	8.52	32.35	18.37
Linolenic, C18:3 ($C_{18}H_{30}O_2$)	2.74	1.71	1.27
Arachidic, C20:0 ($C_{20}H_{40}O_2$)	0.80	5.01	0.02
Gondoic, C20:1 ($C_{20}H_{38}O_2$)	2.11	-	5.70
Behenic, C22:0 ($C_{22}H_{44}O_2$)	-	1.21	1.50
Erocic, C22:1 ($C_{22}H_{42}O_2$)	8.10	-	
Lignoceric, C24:0 ($C_{24}H_{48}O_2$)	-	-	2.40
Nervonic, C24:1 ($C_{24}H_{46}O_2$)		-	-
Pentacosylic, C25:0 ($C_{25}H_{50}O_2$)	-		0.10
Total	100	100	100

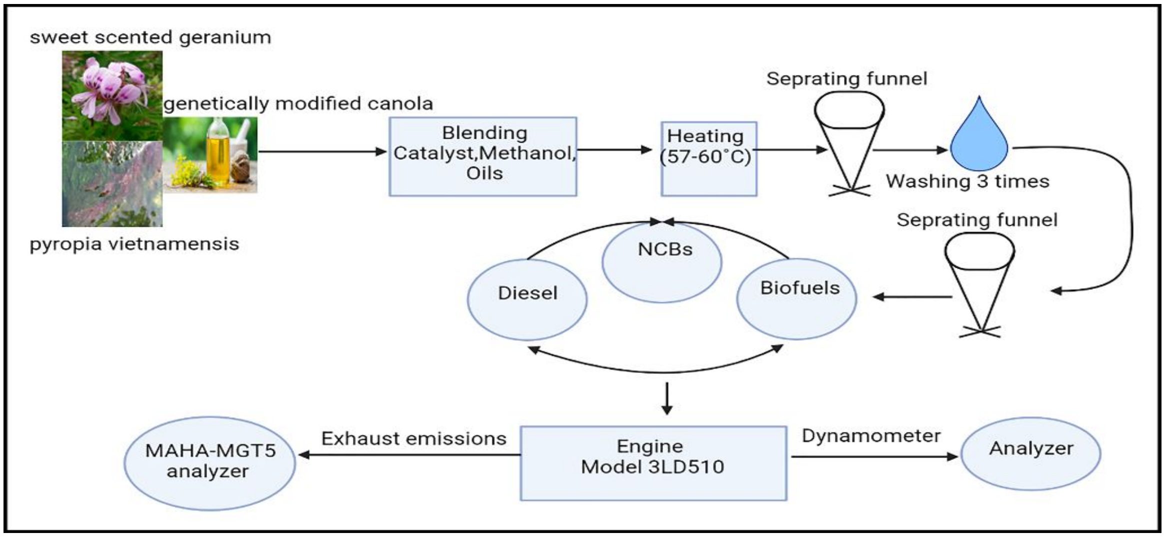

Figure S1. A schematic diagram for biodiesel production and engine test

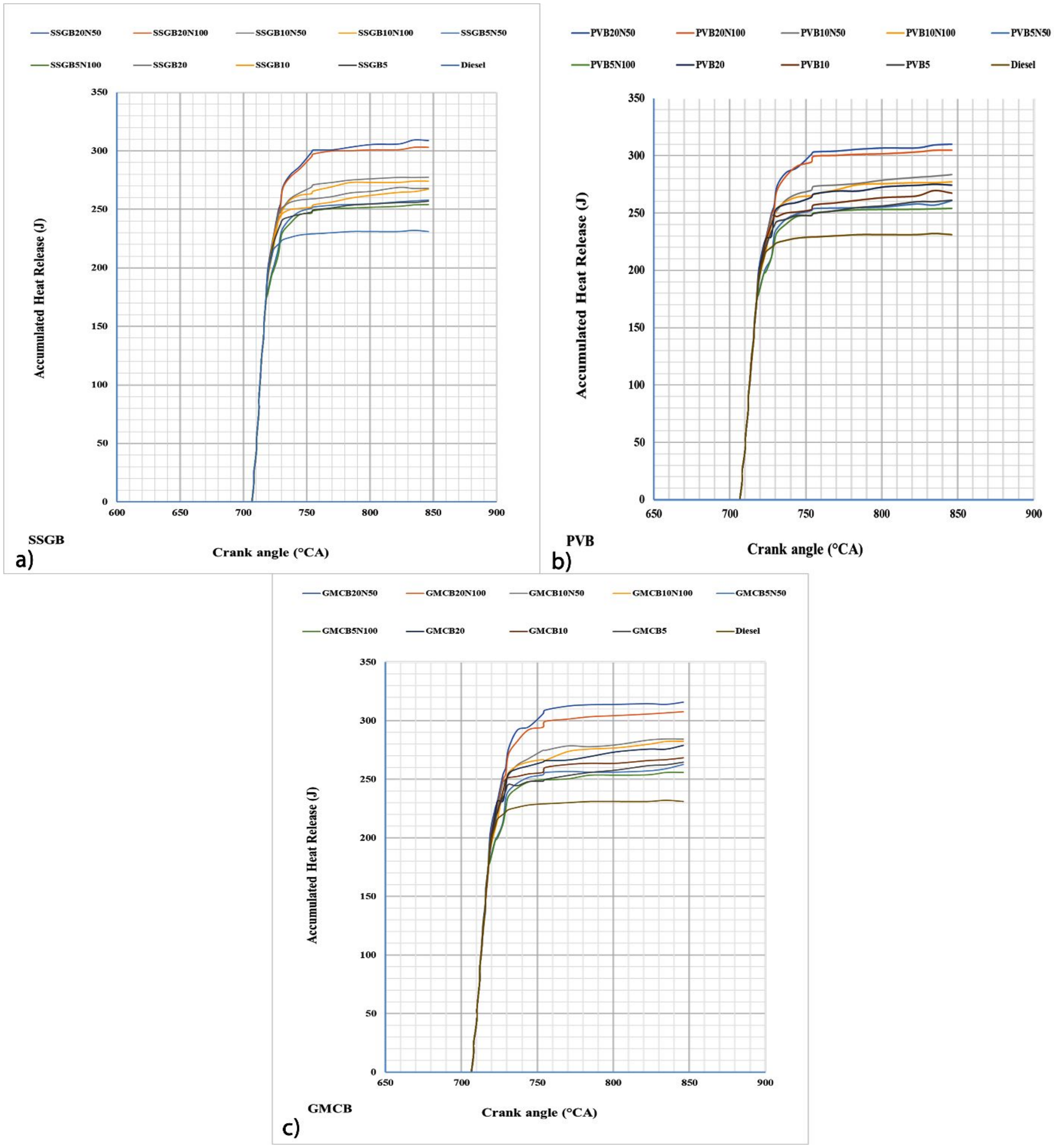

Figure S2. The level of AHR for different level of biodiesel and nano additive for (a): Sweet-scented geranium biodiesel (SSGB), (b): Pyropia vietnamensis biodiesel (PVB), (c): Genetically modified canola biodiesel (GMCB).

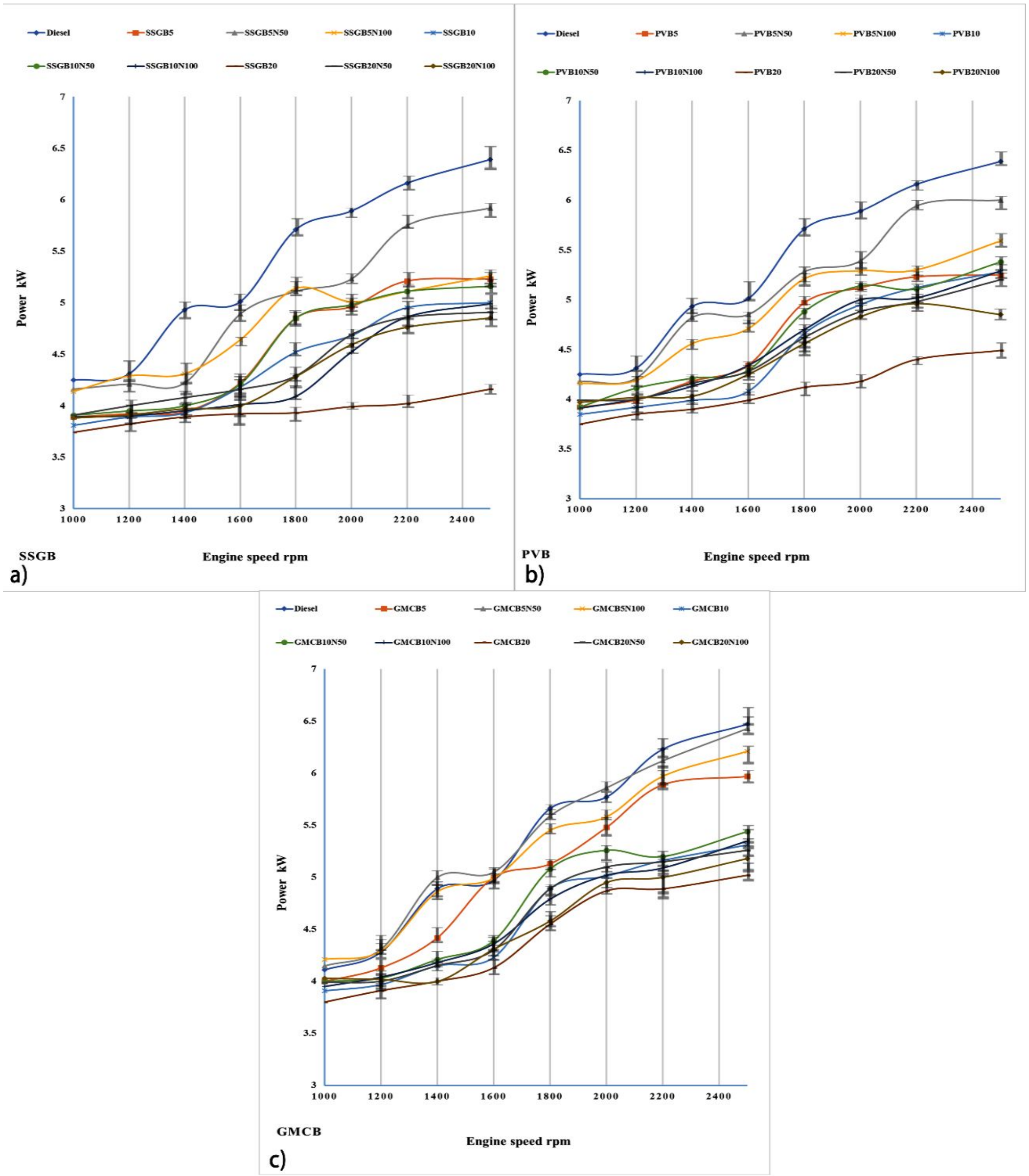

Figure S3. The level of power for different level of biodiesel and nano additive for (a): Sweet-scented geranium biodiesel (SSGB), (b): Pyropia vietnamensis biodiesel (PVB), (c): Genetically modified canola biodiesel (GMCB).

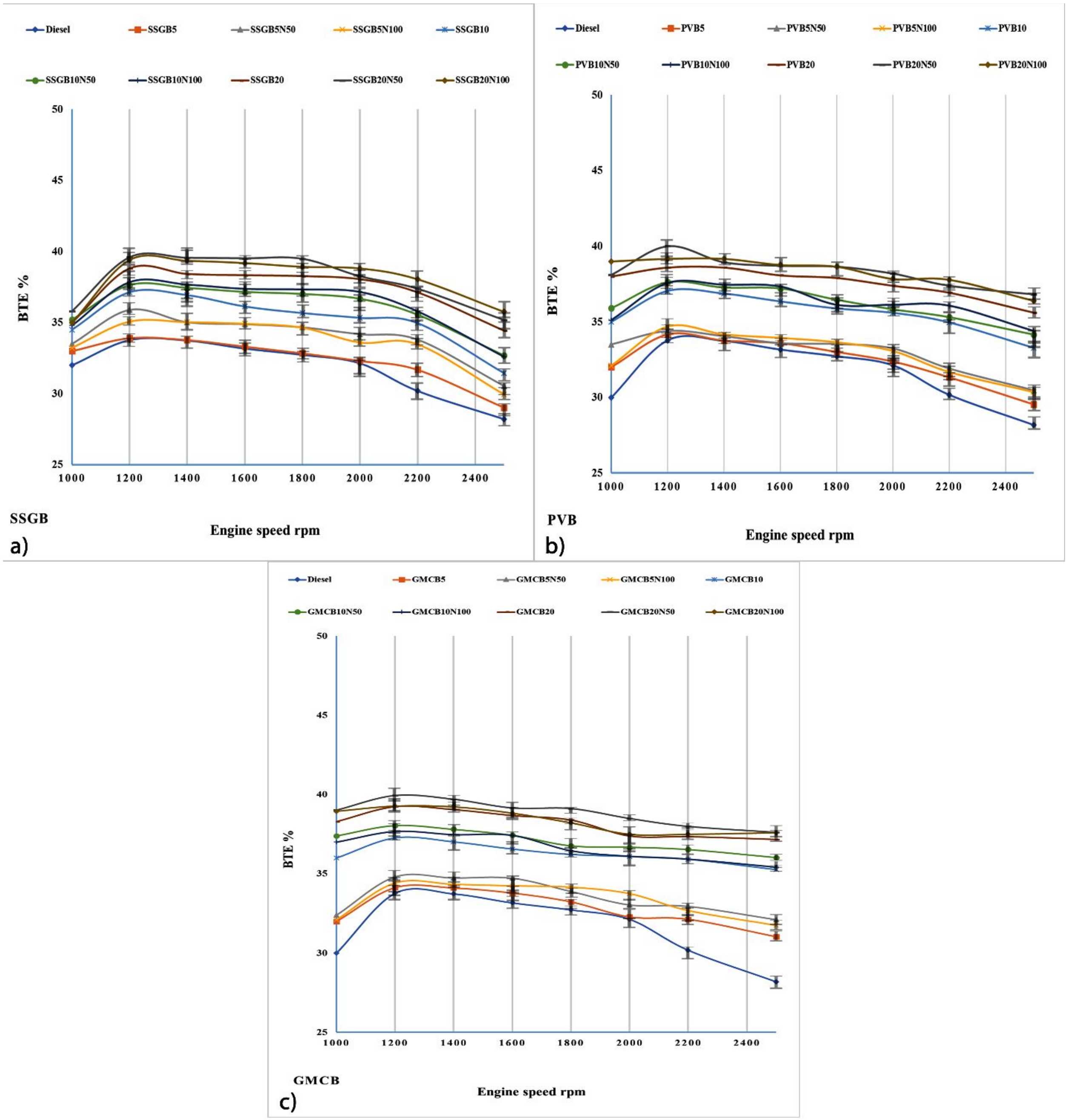

Figure S4. The level of BTEs for different level of biodiesel and nano additive for (a): Sweet-scented geranium biodiesel (SSGB), (b): Pyropia vietnamensis biodiesel (PVB), (c): Genetically modified canola biodiesel (GMCB).

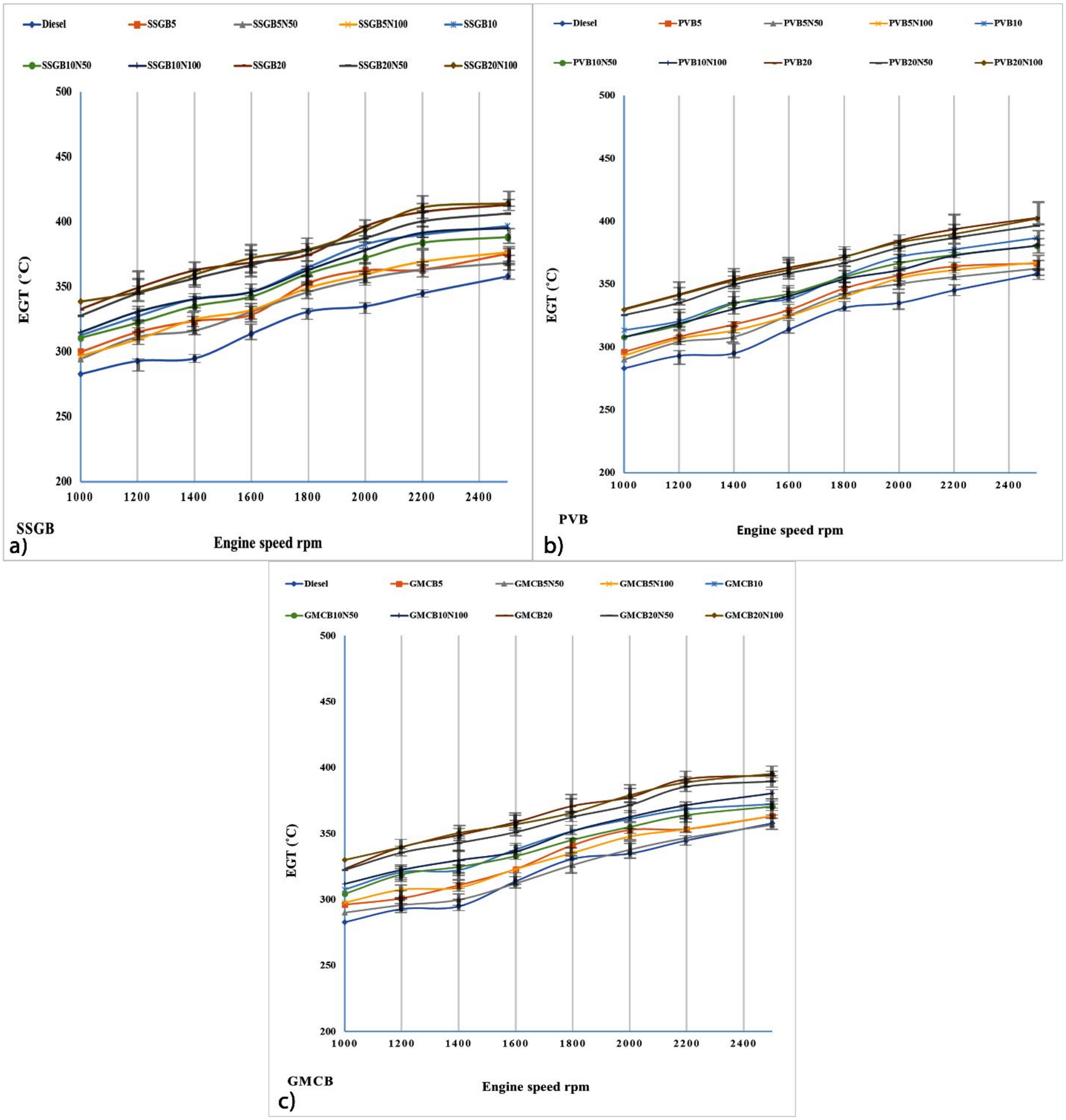

Figure S5. The level of EGTs for different level of biodiesel and nano additive for (a): Sweet-scented geranium biodiesel (SSGB), (b): Pyropia vietnamensis biodiesel (PVB), (c): Genetically modified canola biodiesel (GMCB).

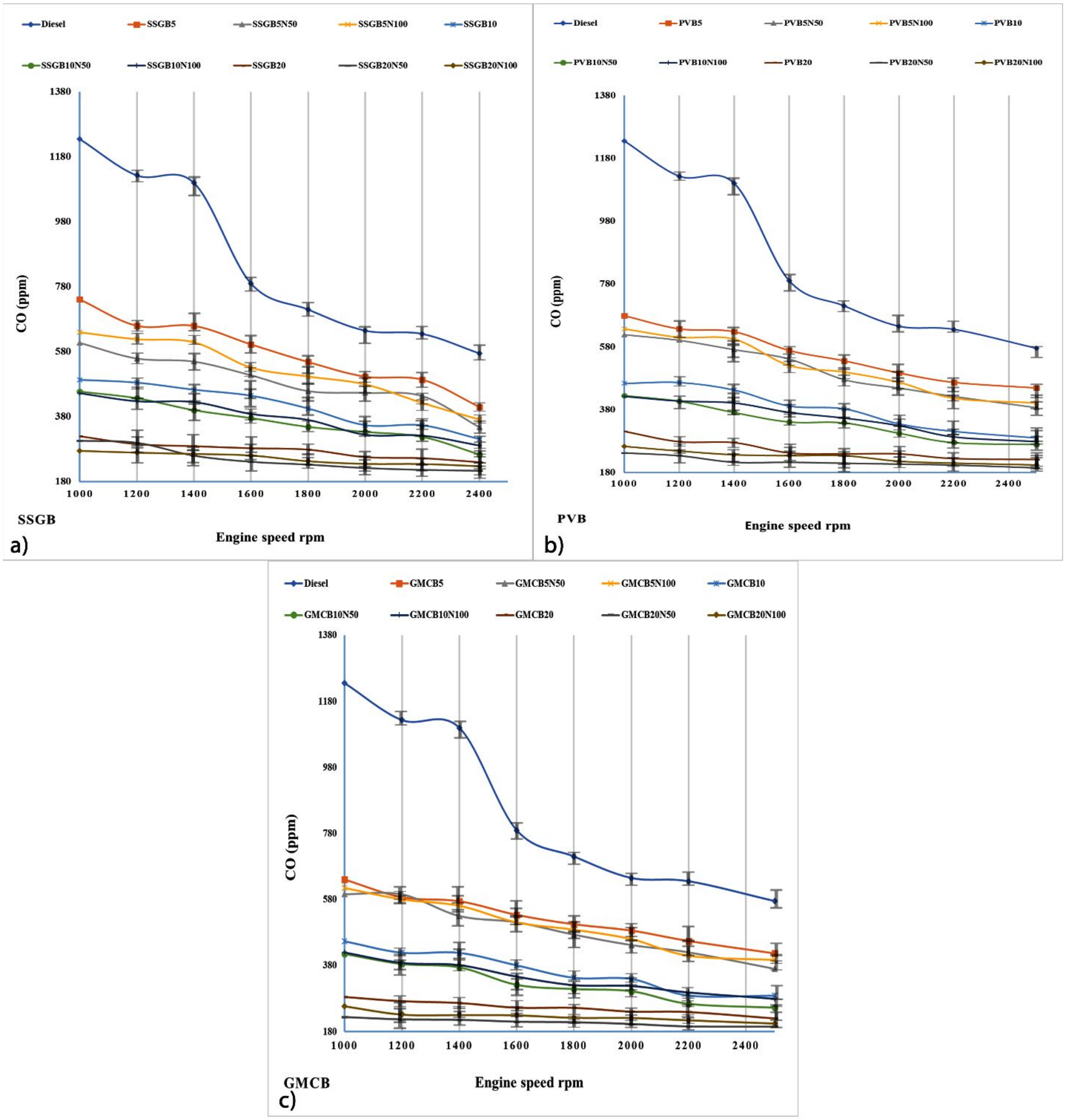

Figure S6. The level of CO for different level of biodiesel and nano additive for (a): Sweet-scented geranium biodiesel (SSGB), (b): Pyropia vietnamensis biodiesel (PVB), (c): Genetically modified canola biodiesel (GMCB).
